# Socio-Economic Factors Impact US Dietary Exposure to Halogenated Flame Retardants

**DOI:** 10.1021/acs.estlett.3c00224

**Published:** 2023-05-17

**Authors:** Yulong Ma, Kevin Andrew Romanak, Staci Lynn Capozzi, Chunjie Xia, Daniel Crawford Lehman, Stuart Harrad, Reginald Cline-Cole, Marta Venier

**Affiliations:** † School of Geography, Earth, and Environmental Sciences, University of Birmingham, Birmingham B15 2TT, U.K.; ‡ O’Neill School of Public and Environmental Affairs, Indiana University, Bloomington, Indiana 47405, USA; § Department of African Studies & Anthropology, School of History and Cultures, University of Birmingham, Birmingham B15 2TT, U.K.

**Keywords:** PBDEs, NBFRs, deca-BDE, bromobenzene, dietary intake, health risk

## Abstract

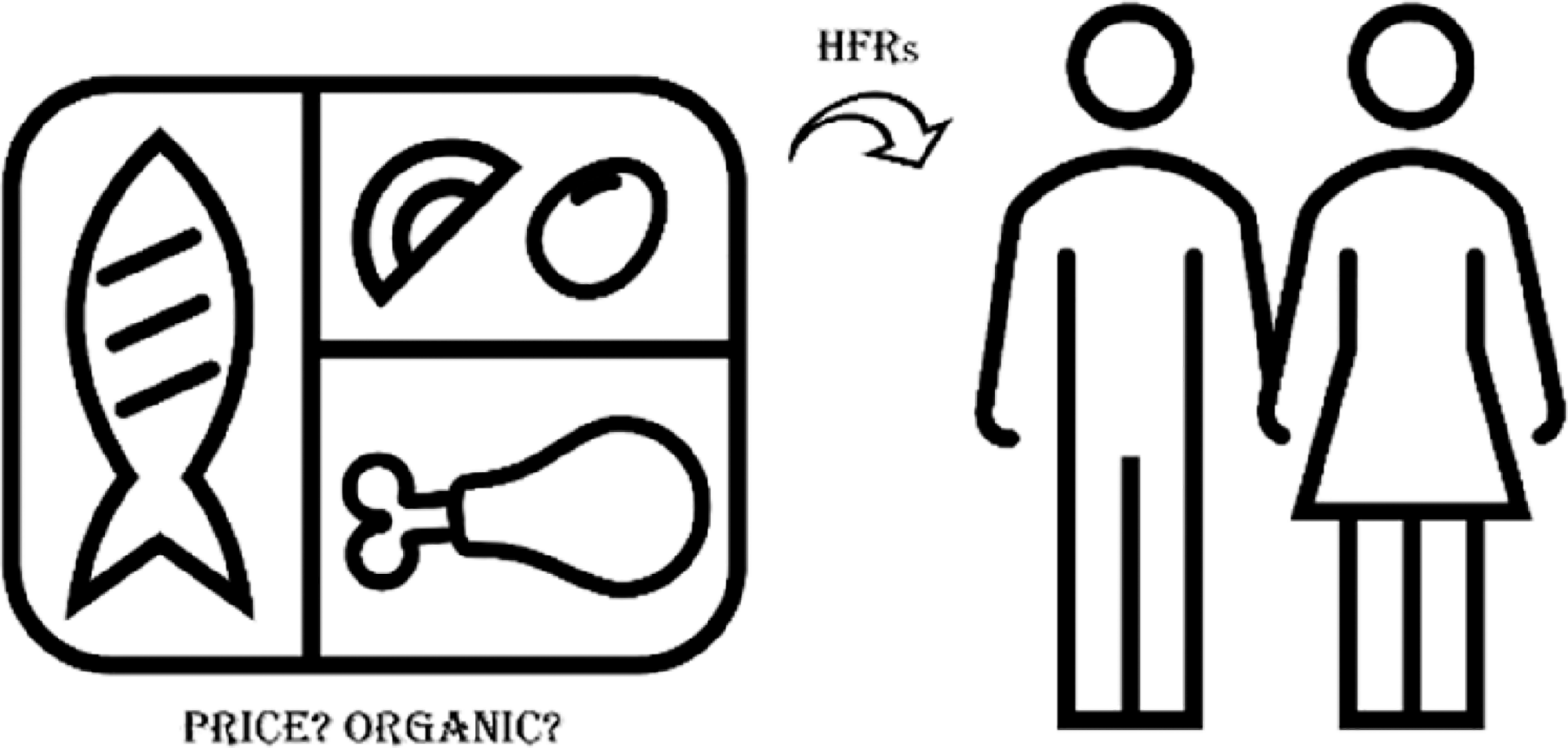

Although diet is an important route of exposure for brominated flame retardants (BFRs), little is known of their presence in US food. Therefore, we purchased meat, fish, and dairy product samples (*n* = 72) in Bloomington, IN, from 3 stores representing national retail chains at different price levels. Composite samples (*n* = 42) were analyzed for polybrominated diphenyl ethers (PBDEs), hexabromocyclododecane (HBCDD), novel BFRs (NBFRs), and dechlorane plus (DP). Concentrations of total halogenated flame retardants (HFRs) ranged between 54 and 1,400 pg/g ww, with PBDEs being the predominant compounds. Concentrations of NBFRs, but not PBDEs, in US food items were significantly impacted by price, raising the issue of environmental justice. Nonorganic food generally had a higher abundance of BDE-209 than organic food items. Estimates of dietary exposure revealed that meat and cheese consumption contribute most to the overall HFR intake and that intakes are highest for children and for non-Hispanic Asians. Taking into account several caveats and limitations of this study, these results as a whole suggest that health burdens from dietary exposure to HFRs have become minimal for US citizens, highlighting the positive impact of regulatory efforts.

## Introduction

Halogenated flame retardants (HFRs) have been extensively used in commercial products, such as electronic and electrical goods, textiles and fabrics, foam for furnishings, and building insulation materials, to help meet fire safety regulations. Polybrominated diphenyl ethers (PBDEs) and hexabromocyclododecane (HBCDD) are two classes of brominated flame retardants (BFRs) which have been widely produced, with their global historical production volumes estimated to reach 1,900,000 and 600,000 tonnes, respectively.^1,2^ Owing to their extensive use, PBDEs and HBCDD have become ubiquitous in the environment, in biota, and in humans.^3−6^ Because of concerns about their adverse impacts on environmental and ecological safety and human health,^7−9^ combined with their persistence in the environment and capacity for bioaccumulation,^10−14^ restrictions on their production and use were introduced. In the US, commercial penta- and octa-BDE mixtures were banned by 2006, with deca-BDE restricted in 2008,^4^ and these commercial mixtures were also listed under the Stockholm Convention on Persistent Organic Pollutants (POPs) in 2004 and 2019, respectively, resulting in a global phase-out of PBDEs.^15^ In the meantime, the global phase-out of these legacy BFRs generated an increased demand for alternative products such as novel BFRs (NBFRs).^4^ Dechlorane plus (DP) was also introduced as a possible replacement for deca-BDE, resulting in a rise in global demand for DP.^16^ As a result of these replacement trends, several alternative HFRs have been frequently detected in the environment and biota in recent years.^17−21^

Despite the extensive use of these legacy and emerging HFRs and their ubiquity, limited information is available on their presence in US food items. An early study investigated concentrations of 43 PBDEs in salmon samples collected throughout the US between September 2001 and December 2002, reporting mean concentrations of Σ_43_ PBDEs of 56–3,300 pg/g ww.^22^ Concentrations of PBDEs and HBCDDs were also observed in individual and composite food samples collected in Dallas, TX, USA, between 2003 and 2010.^23−27^ Another study reported NBFR and DP concentrations in US baby food in 2013.^28^ To the best of our knowledge, recent data on concentrations of these legacy and emerging HFRs in general food items is not available, and an update of the overall trends is needed 10–20 years after these levels were first investigated.

Therefore, the aims of this study were to (1) provide data on current concentrations and relative abundance of legacy and emerging HFRs in US food items; (2) identify impacts of food price on HFR concentrations in US food items; (3) investigate whether there are differences in contamination of these HFRs in organic and nonorganic US food items; and (4) estimate dietary exposure to these HFRs and evaluate any potential health risks, especially in relation to socio-economic factors.

## Materials and Methods

### Sampling

US food samples were purchased in Bloomington, IN, USA, from 3 grocery stores representing national retail chains and processed at Indiana University in Bloomington, IN, USA. The same type of food items were collected from 3 supermarkets representing low (11 composite samples), medium (17 composite samples), and high (14 composite samples) food prices (for example, samples of salmon at the three different price points were purchased). Briefly, a total of 72 individual food samples representing 11 food items, organic and nonorganic, were purchased between March and May 2022. One to three individual samples of each food item were homogenized into one composite sample, generating 42 composite food samples. All composite samples were freeze-dried and then stored at −80 °C before analysis. Detailed information on the food samples is shown in Table S1.

### Analytical Protocols

Chemicals and reagents used in this study are summarized in the Supporting Information. The concentrations of 21 PBDEs, 8 NBFRs, HBCDD, and DP (*syn*-DP and *anti*-DP) were measured in these food samples. Sample extraction and cleanup followed a previously published protocol with minor modifications.^12^ Detailed information on sample extraction and cleanup as well as lipid content determination is given in the Supporting Information. Analyses were performed on an Agilent 7890A gas chromatograph coupled with an Agilent 5975C mass spectrometer (GC/MS) operated in electron capture negative ionization (ENCI) mode. Detailed information has been previously published^29^ and is summarized in Table S2.

### QA/QC

Linearity was obtained from an 11-point calibration for all target compounds (Table S3). The limit of detection (LOD) for each analyte was calculated based on a signal-to-noise ratio of 3 (Table S4). Together with each batch of 8–12 samples, 2 method blanks and 2 matrix spikes (with a known amount of target compounds prepared in hexane) were included (Tables S5 and S6). More information on QA/QC is available in the Supporting Information.

### Estimation of Daily Dietary Intake of HFRs

Daily dietary intake (DI) of HFRs was estimated with the equation below
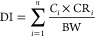
1where *C*_*i*_ is the median concentration (ng/g ww) of HFRs in a particular food item *i*, CR_*i*_ is the average daily food consumption (g/day) of a particular food item *i*, and BW is the average body weight (kg) of US children (<20 years old) and adults (≥20 years old). Further details can be found in the Supporting Information.

### Statistical Analysis

Statistical analysis was done with Excel (Microsoft Office 365) and SPSS Statistics 29.0 (IBM, Chicago, IL, USA). Only the target HFRs with a DF exceeding 30% were included in the analyses. Data were logarithmically transformed, and normality was confirmed using a Shapiro–Wilk test. Blanks were subtracted from samples on a mass basis for each batch. For statistical analyses, concentrations below LOD were designated as DF × LOD when DF exceeded 50% for a specific analyte, while concentrations below LOD were designated as zero when 30% < DF < 50%. HFRs detected in less than 30% of our food samples were included in the calculation of totals, but they were excluded from statistical analyses and human dietary exposure estimation.

## Results and Discussion

### Concentrations and Relative Abundance of HFRs in US Food Items

Table 1 summarizes median concentrations of target HFRs in US food items, and Figure S1 depicts the relative contribution of these HFRs in US food. Averages and ranges of HFR concentrations are given in Table S7.

**Table 1:** Median Concentrations (pg/g ww) of HFRs in US Food Items (n.d. = not detected; only HFRs with a detection frequency (DF) exceeding 30% are included)

		Meat				Fish					Dairy		
HFRs	DF (%)	Beef	Pork	Chicken	Turkey	Salmon	Cod	Tilapia	Tuna	Catfish	Cheese	Egg	All
BDE-15	83	51	110	20	75	130	11	75	42	97	210	10	78
BDE-17	71	13	3.9	4.3	2.7	120	n.d.	3.1	n.d.	11	120	4.0	4.3
BDE-28	79	2.9	9.8	10	6.3	92	2.6	13	65	1.6	11	2.8	6.0
BDE-49	52	n.d.	6.4	6.0	4.8	11	3.4	n.d.	12	3.7	13	n.d.	5.3
BDE-47	33	n.d.	n.d.	n.d.	5.1	44	n.d.	n.d.	7.2	13	1.4	n.d.	n.d.
BDE-100	76	3.4	5.4	7.6	3.1	7.7	5.1	0.75	10	7.8	9.3	2.7	5.1
BDE-99	45	n.d.	n.d.	0.29	9.7	1.5	n.d.	n.d.	1.1	6.2	2.7	22	n.d.
BDE-154	88	8.2	11	9.6	8.9	12	8.5	7.0	11	11	32	6.9	9.1
BDE-153	60	2.4	3.4	5.9	7.0	4.8	1.5	n.d.	1.8	5.5	4.8	n.d.	2.8
BDE-139	33	11	n.d.	16	n.d.	n.d.	n.d.	n.d.	n.d.	n.d.	n.d.	4.5	n.d.
BDE-140	48	6.9	7.6	1.2	8.0	n.d.	8.9	n.d.	18	8.3	n.d.	n.d.	n.d.
BDE-183	36	n.d.	4.1	2.1	0.77	n.d.	1.0	n.d.	n.d.	5.6	n.d.	n.d.	n.d.
BDE-209	60	3.7	6.5	9.7	15	3.2	0.55	n.d.	3.1	22	n.d.	4.2	4.6
**Σ**_**13**_**PBDEs**		**130**	**190**	**120**	**200**	**390**	**49**	**110**	**170**	**190**	**510**	**150**	**180**
pTBX	40	n.d.	n.d.	4.2	n.d.	21	2.1	2.6	41	n.d.	n.d.	0.65	n.d.
PBBz	79	2.9	1.5	1.9	n.d.	5.9	2.4	0.84	1.2	5.4	2.9	1.7	2.1
EH-TBB	36	n.d.	7.2	n.d.	n.d.	n.d.	2.4	3.4	n.d.	4.7	n.d.	n.d.	n.d.
BEH-TEBP	33	n.d.	n.d.	0.29	3.8	n.d.	n.d.	4.7	n.d.	1.4	n.d.	0.55	n.d.
**Σ**_**4**_**NBFRs**		**4.4**	**8.7**	**21**	**17**	**44**	**9.5**	**19**	**43**	**12**	**6.6**	**4.5**	**14**
**Σ**_**2**_**DP**		**n.d.**	**n.d.**	**2.1**	**1.0**	**n.d.**	**0.60**	**0.32**	**0.17**	**16.3**	**0.064**	**n.d.**	n.d.

#### PBDEs

PBDEs were the dominant and most detected HFRs in these samples, with an average contribution of 81% to Σ_32_ HFRs. Cheese had the highest median concentration of Σ_21_ PBDEs, followed closely by salmon. These high PBDE concentrations are likely due to the relatively high lipid contents of salmon (8.4%) and cheese (27%) compared to lean meats such as chicken or turkey (∼4%).

BDE-15 was the most detected and predominant PBDE congener in US food items, with an average contribution of 35% to Σ_21_ PBDEs. BDE-17 and BDE-28 accounted on average for 10% and 9.8% to Σ_21_ PBDEs. In contrast, deca-BDE was only detected with an average abundance of 6.8%. The higher abundance of lower-brominated PBDE congeners is likely due to the debromination of high-brominated PBDEs either abiotically in the environment (i.e., photodebromination) or *in vivo* in the animals before they are sacrificed for the market. The pattern is also a reflection of the phase-out of commercial PBDE mixtures in the US.

PBDE concentrations observed in this study were generally similar to what was reported from another two US-based studies (Table S8) on supermarket-purchased salmon (sampling year: 2001–2002)^22^ and composite US food samples (sampling year: 2009).^24^ The two studies mentioned above are 10+ years old; hence, concentrations seem to be relatively constant with time, despite PBDEs having been withdrawn from the market some 10–15 years ago. However, BDE-47 and BDE-99, containing 4 and 5 bromines, respectively, were the most abundant PBDEs detected in US food in the two studies above,^22,24^ while BDE-15, which contains only two bromines, was the predominant PBDE congener observed in this study. Such a difference possibly indicates more advanced aging of PBDEs in US food during the past decade. A recent survey of the UK market using samples collected in 2021 showed that levels of PBDEs had fallen compared to 2015,^30^ indicating a slower response in dietary products to restrictions on use of PBDEs in the US compared to the UK. Concentrations measured in our study were also broadly comparable to those in food items from Europe^30−36^ and Japan,^37^ but they were considerably lower than the concentrations in food items from Tanzania^38^ and China.^13,39^

#### NBFRs

Compared to PBDEs, NBFRs were less abundant in US food, contributing on average 16% to Σ_32_ HFRs. The median concentration of Σ_8_ NBFRs was 21 pg/g ww. The highest concentrations were observed in turkey samples, with a median Σ_8_ NBFR of 110 pg/g ww. This was followed by salmon and pork, with median concentrations of Σ_8_ NBFRs of 78 and 70 pg/g ww, respectively.

In general, pentabromobenzene (PBBz) was the most detected and most abundant NBFR observed in US food, with an average contribution of 32% to Σ_8_ NBFRs. PBBz was particularly abundant in beef and cheese, contributing 48% and 44%, respectively, to Σ_8_ NBFRs. 2,3,5,6-Tetrabromo-p-xylene (pTBX) was also frequently detected with an average contribution of 20% to Σ_8_ NBFRs, and it was particularly abundant in tuna, contributing about 85% to Σ_8_ NBFRs. It is hard to pinpoint sources of bromobenzenes as they were never used in commercial mixtures, but they could be byproducts or degradation products of BDE-209 or decabromodiphenyl ethane (DBDPE).^40,41^

Data on NBFR concentrations in US food is rather scarce, with only one publication reporting similar levels of PBBz, hexabromobenzene (HBBz), and DBDPE in US baby food from 2013 including formula, cereal, and puree (Table S9).^28^ Comparable concentrations of PBBz, pentabromoethylbenzene (PBEB), HBBz, 2-ethyl hexyl-2,3,4,5-tetrabromobenzoate (EH-TBB or TBB), and DBDPE were also reported in UK food items collected in 2020–2021, but concentrations of 1,2-bis(2,4,6-tribromophenoxy) ethane (BTBPE or TBE) and bis(2-ethyl hexyl) tetrabromophthalate (BEH-TEBP or TBPH) in UK samples were considerably higher.^30^ We speculated such differences could be due to differences in consumption of BTBPE and BEH-TEBP in the UK and the US. However, data is very scarce on production and consumption of these two NBFRs, and our speculations require further verification. Further, NBFR concentrations reported in the present study were similar to those observed in food items from France,^32^ Latvia,^14^ Belgium,^34^ China,^42,43^ and Tanzania,^38^ but they were significantly lower than those from e-waste sites in China.^10,39,44^

#### HBCDD

Concentrations of HBCDD in different food items were not shown in Table 1 due to its low detection frequency (17%). However, HBCDD seemed to be particularly abundant in salmon, with a median concentration of 57 pg/g ww.

#### DP

DP (sum of *syn*- and *anti*-DP) was detected in US food samples with a median concentration of 0.18 pg/g ww. The relative contribution of DP to Σ_32_ HFRs was minimal (0.87% on average). Concentrations of DP observed in the present study were comparable to those determined in food items from Japan^37,45^ but were lower than those from Lebanon,^46^ Latvia,^14^ and Belgium (Table S11).^34^

The fraction of *anti*-DP (*f*_anti_), defined as *anti*-DP/total DP, provides useful information about the degradation processes and sources, as *f*_anti_ is approximately 0.75 in commercial mixtures.^20^ In the present study, the mean *f*_anti_ was 0.69 ± 0.24, which is slightly lower than the *f*_anti_ value of 0.75 for commercial DP mixtures.^20^ This was likely due to specific enrichment in *syn*-DP in biota,^46^ as *syn*-DP is more bioaccumulative than *anti*-DP, especially in aquatic species.^20^

### Impact of Price on HFR Concentrations

No statistically significant difference (*p* = 0.830) was shown for Σ_13_ PBDE concentrations in US food items from the three price groups (Table S12), which is possibly due to either a lack of impact of food price or a sample size too small to detect any differences. However, one-way ANOVA revealed a marginally statistically significant difference (*p* = 0.052) in Σ_4_ NBFR concentrations in US food items from the three price groups. Specifically, Σ_4_ NBFR concentrations in food items from the low-price group significantly exceeded those from both the medium-price group (*p* = 0.049) and the high-price group (*p* = 0.021), while the difference between the medium-price group and the high-price group (*p* = 0.615) was not statistically significant.

**Figure 1:**
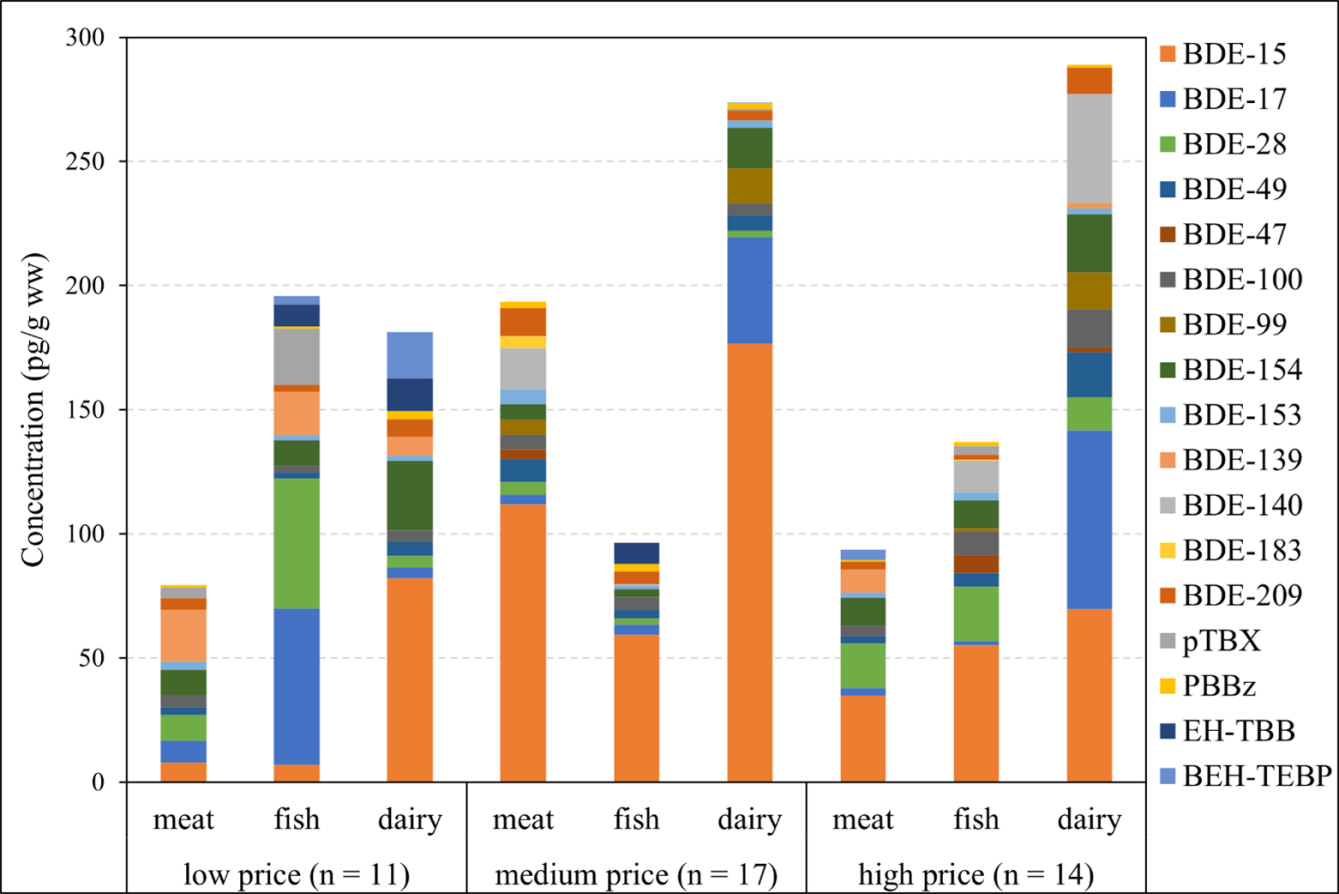
Median concentrations of PBDEs and NBFRs in different US food items of low, medium, and high price. Only PBDEs and NBFRs with a DF exceeding 30% are included.

NBFRs were generally more abundant in low price than in food of medium and high price (Figure 1). Interestingly, the ratios of BDE-209 to low-brominated PBDEs (i.e., BDE-15, BDE-17, and BDE-28) were considerably higher in meat and dairy products of low price than in meat and dairy products of medium and high price. This implied that deca-BDE was phased out earlier in the mass production of higher cost meat and dairy products. Consistent with this hypothesis, the higher ratios of BDE-209 to low-brominated PBDEs were not observed in low-priced fish, possibly because farm raised fish does not involve as much machinery as the production of meat and dairy products.

These results suggest that food price has a minimal impact on supermarket-purchased food items in the US for PBDEs but a significant impact for NBFRs. We speculate that the phase-out of PBDEs in the US drastically reduced PBDE contamination during production, packaging, transportation, and storage of food. On the contrary, ongoing use of NBFRs continues to result in significant contamination in US food and still poses a potential risk for consumers, especially for those purchasing food at the low price point, which raises the issue of environmental justice.

### HFR Concentrations in Organic and Nonorganic Food Items

Median concentrations of Σ_13_ PBDEs and Σ_4_ NBFRs in organic food samples (14 composite samples) were very similar to the corresponding median concentrations in nonorganic food items (15 composite samples; Table S13), with no statistically significant differences observed using paired-samples *t* tests (Σ_13_ PBDEs, *p* = 0.941; Σ_4_ NBFRs, *p* = 0.735).

A relatively higher abundance of BDE-209 but lower abundance of low-brominated PBDEs to total PBDEs was observed in nonorganic US food items compared to organic US food (Figure S2). Such differences were similar to those observed in low-price versus medium- and high-price food and again were likely due to differences between market sectors in the timing of the phase-out of deca-BDE in the US.

### Estimation of Daily Dietary Exposure to HFRs for the US Population

Under a median-exposure scenario, daily dietary intake of HFRs was 0.77 ng/kg bw/day for US children and 0.47 ng/kg bw/day for US adults, respectively. PBDEs constituted 96% of dietary intake of HFRs, with the remaining 4% attributed to NBFRs and DP. Consumption of meat and cheese contributed most to dietary exposure to HFRs, accounting for 57% and 33%, respectively. This was followed by consumption of fish (6%) and chicken eggs (4%).

**Figure 2:**
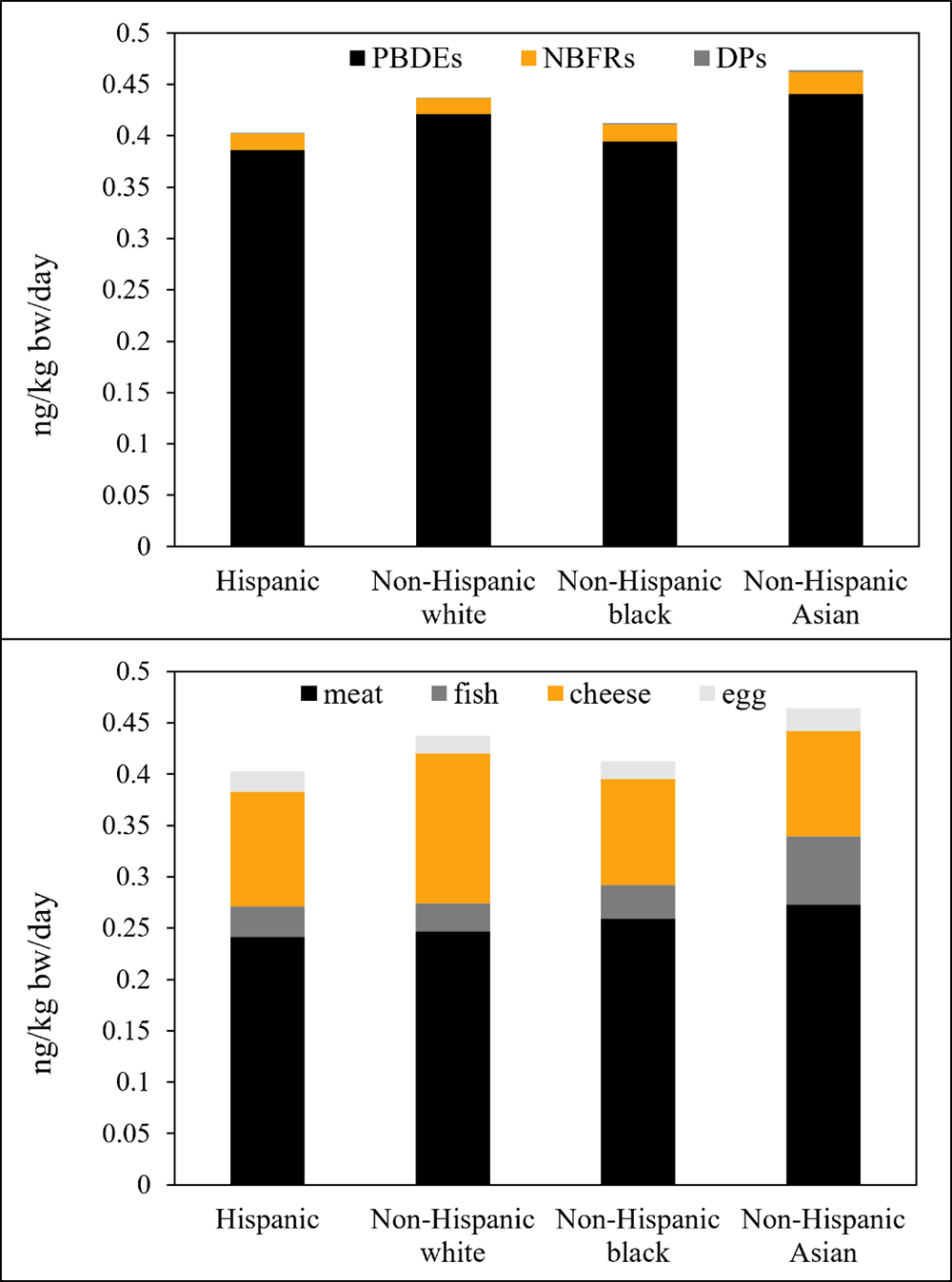
Estimated median dietary intake of HFRs for US adults (≥20 years old) of different races: contribution of different FR classes (upper) and food categories (lower). See Table S19 for detailed data.

Daily dietary intake of HFRs was also estimated for US adults (≥20 years old) of different races (Figure 2 and Table S19). Interestingly, non-Hispanic Asian adults had relatively higher dietary intake of HFRs than did other adult groups. Since non-Hispanic Asians consume a diet richer in fish than other groups, they are likely to ingest more HFRs via fish consumption. Similarly, due to a diet richer in cheese, non-Hispanic white adults were estimated to ingest more HFRs than other groups via cheese intake, which resulted in this group having the second highest dietary intake of HFRs. Due to the limited sample size of this study and the availability of data on food consumption in the US, a more in-depth analysis of the role of socio-economic factors in the overall exposure to HFRs was not possible, but it should be explored in follow-up studies.

Here, the estimated dietary intake of PBDEs for the US population was at least 3 orders of magnitude lower than the corresponding reference doses (RfDs) suggested by the US EPA for all routes of exposure (see Table S20 for a summary of RfDs for HFRs of interest)^47^ and 6–8 orders of magnitude lower than the corresponding RfDs suggested by European Chemicals Bureau^48^ and by two other studies.^49,50^ It is therefore reasonable to speculate that health burdens posed by dietary intake of HFRs are minimal for the US population, although it should be noted that these oral RfDs refer to all possible routes of exposure to HFRs and diet is only one of them, with inhalation and dust ingestion playing a major role. Additionally, the RfD values are available for only a few compounds and do not consider the cumulative effect of complex mixtures containing up to a hundred individual chemicals. Therefore, the contribution of diet in the overall exposure to HFRs should be further evaluated in a broader context. Additionally, these results suggest that some groups of the US population might be exposed to higher levels of HFRs, in particular NBFRs, an observation that should be taken into account in exposure scenarios.

## Supporting Information Available

Additional experimental details, materials, methods, and statistics (PDF)

## References

[ref1] Abbasi G.; Li L.; Breivik K. Global Historical Stocks and Emissions of PBDEs. Environ. Sci. Technol. 2019, 53 (11), 6330–6340. 10.1021/acs.est.8b07032. 31083912

[ref2] Li L.; Wania F. Elucidating the Variability in the Hexabromocyclododecane Diastereomer Profile in the Global Environment. Environ. Sci. Technol. 2018, 52 (18), 10532–10542. 10.1021/acs.est.8b03443. 30146881

[ref3] Ma Y.; Stubbings W. A.; Cline-Cole R.; Harrad S. Human exposure to halogenated and organophosphate flame retardants through informal e-waste handling activities - A critical review. Environ. Pollut. 2021, 268, 115727 10.1016/j.envpol.2020.115727. 33010546

[ref4] Ma Y.; Stubbings W. A.; Abdallah M. A. E.; Cline-Cole R.; Harrad S. Formal waste treatment facilities as a source of halogenated flame retardants and organophosphate esters to the environment: A critical review with particular focus on outdoor air and soil. Sci. Total Environ. 2022, 807, 150747 10.1016/j.scitotenv.2021.150747. 34619188

[ref5] Klincic D.; Dvorscak M.; Jagic K.; Mendas G.; Romanic S. H. Levels and distribution of polybrominated diphenyl ethers in humans and environmental compartments: a comprehensive review of the last five years of research. Environ. Sci. Pollut Res. 2020, 27 (6), 5744–5758. 10.1007/s11356-020-07598-7. 31933075

[ref6] Jiang Y.; Yuan L.; Lin Q.; Ma S.; Yu Y. Polybrominated diphenyl ethers in the environment and human external and internal exposure in China: A review. Sci. Total Environ. 2019, 696, 133902 10.1016/j.scitotenv.2019.133902. 31470322

[ref7] McDonald T. A. A perspective on the potential health risks of PBDEs. Chemosphere 2002, 46 (5), 745–755. 10.1016/S0045-6535(01)00239-9. 11999798

[ref8] Yu L.; Han Z.; Liu C. A review on the effects of PBDEs on thyroid and reproduction systems in fish. Gen comp endocr 2015, 219, 64–73. 10.1016/j.ygcen.2014.12.010. 25585150

[ref9] Update of the risk assessment of hexabromocyclododecanes (HBCDDs) in food. EFSA Journal 2021, 19 (3), e06421 10.2903/j.efsa.2021.6421. 33732387 PMC7938899

[ref10] Labunska I.; Abdallah M. A. E.; Eulaers I.; Covaci A.; Tao F.; Wang M.; Santillo D.; Johnston P.; Harrad S. Human dietary intake of organohalogen contaminants at e-waste recycling sites in Eastern China. Environ. Int. 2015, 74, 209–220. 10.1016/j.envint.2014.10.020. 25454238

[ref11] Fernandes A. R.; Mortimer D.; Rose M.; Smith F.; Panton S.; Garcia-Lopez M. Bromine content and brominated flame retardants in food and animal feed from the UK. Chemosphere 2016, 150, 472–478. 10.1016/j.chemosphere.2015.12.042. 26733012

[ref12] Tao F.; Abdallah M. A. E.; Ashworth D. C.; Douglas P.; Toledano M. B.; Harrad S. Emerging and legacy flame retardants in UK human milk and food suggest slow response to restrictions on use of PBDEs and HBCDD. Environ. Int. 2017, 105, 95–104. 10.1016/j.envint.2017.05.010. 28525835

[ref13] Wang J.; Zhao X.; Wang Y.; Shi Z. Tetrabromobisphenol A, hexabromocyclododecane isomers and polybrominated diphenyl ethers in foodstuffs from Beijing, China: Contamination levels, dietary exposure and risk assessment. Sci. Total Environ. 2019, 666, 812–820. 10.1016/j.scitotenv.2019.02.324. 30818205

[ref14] Zacs D.; Perkons I.; Abdulajeva E.; Pasecnaja E.; Bartkiene E.; Bartkevics V. Polybrominated diphenyl ethers (PBDEs), hexabromocyclododecanes (HBCDD), dechlorane-related compounds (DRCs), and emerging brominated flame retardants (EBFRs) in foods: The levels, profiles, and dietary intake in Latvia. Sci. Total Environ. 2021, 752, 141996 10.1016/j.scitotenv.2020.141996. 33207505

[ref15] Sharkey M.; Harrad S.; Abou-Elwafa Abdallah M.; Drage D. S.; Berresheim H. Phasing-out of legacy brominated flame retardants: The UNEP Stockholm Convention and other legislative action worldwide. Environ. Int. 2020, 144, 106041 10.1016/j.envint.2020.106041. 32822924

[ref16] Chen K.; Zheng J.; Yan X.; Yu L.; Luo X.; Peng X.; Yu Y.; Yang Z.; Mai B. Dechlorane Plus in paired hair and serum samples from e-waste workers: Correlation and differences. Chemosphere 2015, 123, 43–47. 10.1016/j.chemosphere.2014.11.058. 25542638

[ref17] Hou R.; Lin L.; Li H.; Liu S.; Xu X.; Xu Y.; Jin X.; Yuan Y.; Wang Z. Occurrence, bioaccumulation, fate, and risk assessment of novel brominated flame retardants (NBFRs) in aquatic environments - A critical review. Water Res. 2021, 198, 117168 10.1016/j.watres.2021.117168. 33962238

[ref18] McGrath T. J.; Ball A. S.; Clarke B. O. Critical review of soil contamination by polybrominated diphenyl ethers (PBDEs) and novel brominated flame retardants (NBFRs); concentrations, sources and congener profiles. Environ. Pollut. 2017, 230, 741–757. 10.1016/j.envpol.2017.07.009. 28732337

[ref19] Sverko E.; Tomy G. T.; Reiner E. J.; Li Y. F.; McCarry B. E.; Arnot J. A.; Law R. J.; Hites R. A. Dechlorane Plus and Related Compounds in the Environment: A Review. Environ. Sci. Technol. 2011, 45 (12), 5088–5098. 10.1021/es2003028. 21574656

[ref20] Feo M. L.; Baron E.; Eljarrat E.; Barcelo D. Dechlorane Plus and related compounds in aquatic and terrestrial biota: a review. Anal Bioanal Chem. 2012, 404 (9), 2625–2637. 10.1007/s00216-012-6161-x. 22695503

[ref21] Ghelli E.; Cariou R.; Dervilly G.; Pagliuca G.; Gazzotti T. Dechlorane Plus and Related Compounds in Food-A Review. Int. J. Environ. Res. Public Health 2021, 18 (2), 690 10.3390/ijerph18020690. 33466958 PMC7830114

[ref22] Hites R. A.; Foran J. A.; Schwager S. J.; Knuth B. A.; Hamilton M. C.; Carpenter D. O. Global Assessment of Polybrominated Diphenyl Ethers in Farmed and Wild Salmon. Environ. Sci. Technol. 2004, 38 (19), 4945–4949. 10.1021/es049548m. 15506184

[ref23] Schecter A.; Szabo D. T.; Miller J.; Gent T. L.; Malik-Bass N.; Petersen M.; Paepke O.; Colacino J. A.; Hynan L. S.; Harris T. R. Hexabromocyclododecane (HBCD) stereoisomers in US food from Dallas, Texas. Environ. Health Perspect 2012, 120 (9), 1260–1264. 10.1289/ehp.1204993. 22647707 PMC3440131

[ref24] Schecter A.; Haffner D.; Colacino J.; Patel K.; Papke O.; Opel M.; Birnbaum L. Polybrominated diphenyl ethers (PBDEs) and hexabromocyclodecane (HBCD) in composite U.S. food samples. Environ. Health Perspect 2010, 118 (3), 357–362. 10.1289/ehp.0901345. 20064778 PMC2854763

[ref25] Schecter A.; Harris T. R.; Shah N.; Musumba A.; Päpke O. Brominated flame retardants in US food. Mol. Nutr Food Res. 2008, 52 (2), 266–272. 10.1002/mnfr.200700166. 18040989

[ref26] Schecter A.; Papke O.; Harris T. R.; Tung K. C.; Musumba A.; Olson J.; Birnbaum L. Polybrominated diphenyl ether (PBDE) levels in an expanded market basket survey of U.S. food and estimated PBDE dietary intake by age and sex. Environ. Health Perspect 2006, 114 (10), 1515–1520. 10.1289/ehp.9121. 17035135 PMC1626425

[ref27] Schecter A.; Päpke O.; Tung K.-C.; Staskal D.; Birnbaum L. Polybrominated Diphenyl Ethers Contamination of United States Food. Environ. Sci. Technol. 2004, 38 (20), 5306–5311. 10.1021/es0490830. 15543730

[ref28] Liu L. Y.; Salamova A.; Hites R. A. Halogenated flame retardants in baby food from the United States and from China and the estimated dietary intakes by infants. Environ. Sci. Technol. 2014, 48 (16), 9812–9818. 10.1021/es502743q. 25084546

[ref29] Ma Y.; Salamova A.; Venier M.; Hites R. A. Has the phase-out of PBDEs affected their atmospheric levels? Trends of PBDEs and their replacements in the Great Lakes atmosphere. Environ. Sci. Technol. 2013, 47 (20), 11457–11464. 10.1021/es403029m. 24059785

[ref30] Ma Y.; Stubbings W. A.; Abdallah M. A. E.; Cline-Cole R.; Harrad S. Temporal trends in concentrations of brominated flame retardants in UK foodstuffs suggest active impacts of global phase-out of PBDEs and HBCDD. Sci. Total Environ. 2023, 863, 160956 10.1016/j.scitotenv.2022.160956. 36528953

[ref31] Riviere G.; Sirot V.; Tard A.; Jean J.; Marchand P.; Veyrand B.; Le Bizec B.; Leblanc J. C. Food risk assessment for perfluoroalkyl acids and brominated flame retardants in the French population: results from the second French total diet study. Sci. Total Environ. 2014, 491–492, 176–183. 10.1016/j.scitotenv.2014.01.104. 24529894

[ref32] Venisseau A.; Bichon E.; Brosseaud A.; Vaccher V.; Lesquin E.; Larvor F.; Durand S.; Dervilly-Pinel G.; Marchand P.; Le Bizec B. Occurence of legacy and novel brominated flame retardants in food and feed in France for the period 2014 to 2016. Chemosphere 2018, 207, 497–506. 10.1016/j.chemosphere.2018.05.122. 29843025

[ref33] Covaci A.; Roosens L.; Dirtu A. C.; Waegeneers N.; Van Overmeire I.; Neels H.; Goeyens L. Brominated flame retardants in Belgian home-produced eggs: levels and contamination sources. Sci. Total Environ. 2009, 407 (15), 4387–4396. 10.1016/j.scitotenv.2008.09.057. 18986684

[ref34] Poma G.; Malysheva S. V.; Goscinny S.; Malarvannan G.; Voorspoels S.; Covaci A.; Van Loco J. Occurrence of selected halogenated flame retardants in Belgian foodstuff. Chemosphere 2018, 194, 256–265. 10.1016/j.chemosphere.2017.11.179. 29216545

[ref35] Garcia Lopez M.; Driffield M.; Fernandes A. R.; Smith F.; Tarbin J.; Lloyd A. S.; Christy J.; Holland M.; Steel Z.; Tlustos C. Occurrence of polybrominated diphenylethers, hexabromocyclododecanes, bromophenols and tetrabromobisphenols A and S in Irish foods. Chemosphere 2018, 197, 709–715. 10.1016/j.chemosphere.2018.01.089. 29407835

[ref36] Trabalon L.; Vilavert L.; Domingo J. L.; Pocurull E.; Borrull F.; Nadal M. Human exposure to brominated flame retardants through the consumption of fish and shellfish in Tarragona County (Catalonia, Spain). Food Chem. Toxicol. 2017, 104, 48–56. 10.1016/j.fct.2016.11.022. 27887975

[ref37] Kakimoto K.; Nagayoshi H.; Yoshida J.; Akutsu K.; Konishi Y.; Toriba A.; Hayakawa K. Detection of Dechlorane Plus and brominated flame retardants in marketed fish in Japan. Chemosphere 2012, 89 (4), 416–419. 10.1016/j.chemosphere.2012.05.072. 22698370

[ref38] Polder A.; Muller M. B.; Brynildsrud O. B.; de Boer J.; Hamers T.; Kamstra J. H.; Lie E.; Mdegela R. H.; Moberg H.; Nonga H. E.; Sandvik M.; Skaare J. U.; Lyche J. L. Dioxins, PCBs, chlorinated pesticides and brominated flame retardants in free-range chicken eggs from peri-urban areas in Arusha, Tanzania: Levels and implications for human health. Sci. Total Environ. 2016, 551–552, 656–667. 10.1016/j.scitotenv.2016.02.021. 26897409

[ref39] Zeng Y. H.; Luo X. J.; Tang B.; Mai B. X. Habitat- and species-dependent accumulation of organohalogen pollutants in home-produced eggs from an electronic waste recycling site in South China: Levels, profiles, and human dietary exposure. Environ. Pollut. 2016, 216, 64–70. 10.1016/j.envpol.2016.05.039. 27239689

[ref40] Venier M.; Ma Y.; Hites R. A. Bromobenzene flame retardants in the Great Lakes atmosphere. Environ. Sci. Technol. 2012, 46 (16), 8653–8660. 10.1021/es3015919. 22849422

[ref41] Ma Y.; Venier M.; Hites R. A. Tribromophenoxy flame retardants in the Great Lakes atmosphere. Environ. Sci. Technol. 2012, 46 (24), 13112–13117. 10.1021/es3033814. 23181569

[ref42] Shi Z.; Zhang L.; Li J.; Zhao Y.; Sun Z.; Zhou X.; Wu Y. Novel brominated flame retardants in food composites and human milk from the Chinese Total Diet Study in 2011: Concentrations and a dietary exposure assessment. Environ. Int. 2016, 96, 82–90. 10.1016/j.envint.2016.09.005. 27619751

[ref43] Jian K.; Zhao L.; Ya M.; Zhang Y.; Su H.; Meng W.; Li J.; Su G. Dietary intake of legacy and emerging halogenated flame retardants using food market basket estimations in Nanjing, eastern China. Environ. Pollut. 2020, 258, 113737 10.1016/j.envpol.2019.113737. 31838397

[ref44] Zheng X.; Xu F.; Luo X.; Mai B.; Covaci A. Phosphate flame retardants and novel brominated flame retardants in home-produced eggs from an e-waste recycling region in China. Chemosphere 2016, 150, 545–550. 10.1016/j.chemosphere.2015.09.098. 26460270

[ref45] Kakimoto K.; Nagayoshi H.; Takagi S.; Akutsu K.; Konishi Y.; Kajimura K.; Hayakawa K.; Toriba A. Inhalation and dietary exposure to Dechlorane Plus and polybrominated diphenyl ethers in Osaka, Japan. Ecotoxicol Environ. Saf 2014, 99, 69–73. 10.1016/j.ecoenv.2013.10.023. 24211159

[ref46] Abdel Malak I.; Cariou R.; Guiffard I.; Venisseau A.; Dervilly-Pinel G.; Jaber F.; Le Bizec B. Assessment of Dechlorane Plus and related compounds in foodstuffs and estimates of daily intake from Lebanese population. Chemosphere 2019, 235, 492–497. 10.1016/j.chemosphere.2019.06.148. 31276863

[ref47] US Environmental Protection Agency (US EPA). Technical Fact Sheet - Polybrominated Diphenyl Ethers (PBDEs) (EPA 505-F-17-015). 2017. Available at: https://www.epa.gov/sites/default/files/2014-03/documents/ffrrofactsheet_contaminant_perchlorate_january2014_final_0.pdf (accessed September 2022).

[ref48] Pakalin S.; Cole T.; Steinkellner J.; Nicolas R.; Tissier C.; Munn S.; Eisenreich S. J. E. R. E. Review on production processes of decabromodiphenyl ether (decaBDE) used in polymeric applications in electrical and electronic equipment, and assessment of the availability of potential alternatives to decaBDE. European Report EUR 2007, 22693, 47.

[ref49] Wang D. G.; Alaee M.; Byer J. D.; Brimble S.; Pacepavicius G. Human health risk assessment of occupational and residential exposures to dechlorane plus in the manufacturing facility area in China and comparison with e-waste recycling site. Sci. Total Environ. 2013, 445–446, 329–336. 10.1016/j.scitotenv.2012.12.059. 23354373

[ref50] Ali N.; Dirtu A. C.; Van den Eede N.; Goosey E.; Harrad S.; Neels H.; t Mannetje A.; Coakley J.; Douwes J.; Covaci A. Occurrence of alternative flame retardants in indoor dust from New Zealand: indoor sources and human exposure assessment. Chemosphere 2012, 88 (11), 1276–1282. 10.1016/j.chemosphere.2012.03.100. 22551874

