# Supplementary material for: Socio-Economic Factors Impact US Dietary Exposure to Halogenated Flame Retardants
Source: Environ Sci Technol Lett. 2023 May 17;10(6):478–84. doi: 10.1021/acs.estlett.3c00224 (PMC10269323; doi:10.1021/acs.estlett.3c00224)
Supplement: Supplementary file 1 — ez3c00224_si_001.pdf [file ez3c00224_si_001.pdf]

Socio-economic factors impact US dietary exposure to halogenated flame retardants

Supporting Information

Yulong Ma [a, b], Kevin Andrew Romanak [b], Staci Lynn Capozzi [b], Chunjie Xia [b], Daniel Crawford Lehman [b], Stuart Harrad [a], Reginald Cline-Cole [c], Marta Venier [b, *]

[a] School of Geography, Earth, and Environmental Sciences,
University of Birmingham,
Birmingham B15 2TT, UK
[b] O'Neill School of Public and Environmental Affairs,
Indiana University,
Bloomington, Indiana 47405, USA
[c] Department of African Studies & Anthropology, School of History and Cultures,
University of Birmingham,
Birmingham, B15 2TT,UK

[*] Corresponding author. E-mail address: mvenier@indiana.edu (M. Venier)

Chemicals and reagents

All the solvents used in this study were purchased from OmniSolv® (Burlington, MA, USA), and were of HPLC analytical grade. Sulfuric acid (95-98%, ACS grade) was purchased from VWR Int. LLC. (Radnor, PA, USA). Potassium Chloride (ACS grade) was purchased from Fisher Scientific (Ottawa, ON, Canada). Alumina was purchased from Fisher Scientific (Ottawa, ON, Canada), and was baked at 300℃ for 8h and then activated with 6% deionized water prior to use. All the native and labelled standards were purchased from Wellington Laboratories (Guelph, ON, Canada), except for BDE-77 (obtained from AccuStandard Inc. (New Haven, CT, USA)) as well as native HBCDDs (obtained from Sigma-Aldrich Company Ltd. (Dorset, UK)).

Sample extraction and clean-up

The concentrations of 21 PBDEs (di-BDE (BDE-15), tri-BDE (BDE-17, -28), tetra-BDE (BDE-49, -47, -66), penta-BDE (BDE-100, -99, -85), hexa-BDE (BDE-154, -153, -139, -140), hepta-BDE (BDE-183), octa-BDE (BDE-201, -197, -203), nona-BDE (BDE-208, -207, -206), and deca-BDE (BDE-209)), 8 NBFRs (2,3,5,6-tetrabromo-p-xylene (pTBX), pentabromobenzene (PBBz), pentabromoethylbenzene (PBEB), hexabromobenzene (HBBz), 2-ethyl hexyl-2,3,4,5-tetrabromobenzoate (EH-TBB or TBB), 1,2-bis(2,4,6-tribromophenoxy) ethane (BTBPE or TBE), bis(2-ethyl hexyl) tetrabromophthalate (BEH-TEBP or TBPH), and decabromodiphenyl ethane (DBDPE)), HBCDD, and DP (*syn*-DP and *anti*-DP) were measured in food samples.

Approximately 0.5 g of freeze-dried food samples were precisely weighted and spiked with 3 ng of BDE-77, 5 ng of BDE-166, and 4 ng of 13C-BDE-209 as surrogate standards before extraction. Hexane/Acetone (1:1, v/v) was used to extract the samples on an accelerated solvent extractor (Dionex ASE 350). The ASE cells (34 mL) were filled from bottom to top with: pre-cleaned hydromatrix, 6 g of alumina, samples, and pre-cleaned hydromatrix. The extraction programme was set as: *Temperature: 100℃; Heating time: 5 min; Static time: 4 min; Cycles: 3; Pressure: 1500 psi. Purge time: 90 s; Flush volume: 60%.* The extracts were collected in pre-cleaned ASE collection bottles, and then were concentrated to 5 mL under a gentle nitrogen stream before being transferred into pre-weighted 15 mL glass tubes. Approximately 10% of the concentrated extracts were transferred into pre-weighted aluminum disks. The aluminum disks were then stored at room temperature overnight before being weighted again. This allowed lipid content of the samples to be determined gravimetrically. The remaining ~90% of the extracts were vortexed with 4 mL KCl

solution (1% in water). Then the samples were centrifuged at 3000 RPM for 5 min, and the upper layer was transferred into another 15 mL glass tube, with the bottom layer extracted with 4 mL hexane twice and the extracts combined. Following this, the extracts were concentrated to 5 mL before shaking with 5 mL sulfuric acid (95%) to remove lipids and proteins. The samples were then centrifuged at 3500 RPM for 20 min, and the solvent phase (upper layer) was collected. The acid phase (bottom layer) was extracted with 5 mL hexane twice and then centrifuged, and the solvents were collected and combined. The purified extracts were concentrated to < 1 mL under a gentle stream of nitrogen, and then were reconstituted into 1 mL hexane containing 5 ng of BDE-118 and 10 ng of BDE-181 as internal standards before GC-MS analysis.

Instrumental analysis

Analyses of BFRs and DPs were performed on an Agilent 7890A gas chromatograph coupled with an Agilent 5975C mass spectrometer (GC/MS) operated in electron capture negative ionization (ENCI) mode. Chromatographic resolution is achieved with a Restek Rtx-1614 (15 m length, 0.25 mm ID, 0.1 μm film thickness) fused silica capillary column (Restek Corporation, Bellefonte, CA) with helium (99.999%; Liquid Carbonic, Chicago, USA) as the carrier gas and methane (99.97%; Praxair, Indianapolis, USA) as the reagent gas. Injections were analysed by GC/MS under selected ion monitoring (SIM) mode, and the quantitation parameters were summarized in Table S2.

QA/QC

Table S3 shows linearity of target analytes and surrogate standards obtained from the 11-point calibration. Limit of detection (LOD) for each analyte was calculated based on a signal/noise ratio of 3, and the data is presented in Table S4. Recoveries of surrogate (internal) standards in all the blanks, matrix spikes, and samples are given in Table S5. Most target analytes were not detected in the blanks, and thus the corresponding samples were not blank-corrected. Only BDE-15, -47, -99, -153, -139, -140, -183, -209, EH-TBB, BEH-TEBP, and anti-DP were detected in at least 1 blank at very low concentrations (slightly higher than the LODs), so the corresponding samples were blank-corrected by subtracting their mass in blanks from the mass detected in samples. Recoveries of the target analytes in matrix spikes are shown in Table S6.

Estimation of daily dietary intake of HFRs. Daily dietary intake of HFRs for the US population were estimated with equation 1. Daily consumption of various food items was calculated from US per capita loss-adjusted food availability,[1] after conversion from lb/year to g/day (Tables

S14-S15). Data on body weight (kg) for the US population is summarized in Tables S16-S17.[2] Median concentrations of HFRs in different food items were used to estimate a median human dietary exposure to HFRs. Fig. S3 shows estimated daily dietary intake of HFRs for US children (< 20 years old) and adults (≥ 20 years old), with detailed data provided in Table S18. Daily dietary intake of HFRs was also estimated for US adults (≥ 20 years old) of different races namely Hispanic (including Mexican-Americans), non-Hispanic white, non-Hispanic black, and non-Hispanic Asian. We recognize that grouping all children in the same category is a limitation that was forced by the availability of data on daily food consumption in the US.

Table S1. Detailed information on US food items analyzed in the present study

**Food items**	**Number of composite samples analyzed**	**Number of individual samples included**	**Water content %**	**Lipid content %**
**Meat**	**18**	**34**	**-**	**-**
beef (organic vs non-organic)	4	9	70	6.3
pork (organic vs non-organic)	4	9	64	12
chicken (organic vs non-organic)	6	11	74	3.5
turkey (organic vs non-organic)	4	5	73	4.3
**Fish**	**13**	**17**	**-**	**-**
Salmon	3	5	69	8.4
Cod	3	4	82	0.52
Catfish	2	3	82	4.5
Tilapia	3	3	80	0.85
Tuna	2	2	73	0.43
**cheese** (organic vs non-organic)	**6**	**6**	**36**	**27**
**egg** (organic vs non-organic)	**5**	**15**	**76**	**5.5**

Table S2. HFR analyte list and quantitation parameters

Compound	Elute Order	Type	Internal Standard	Quant Ion	Confirmation		
					Ion I	Ion II	Ion III
BDE-15	1	Target	BDE-118	81	79		
pTBX	2	Target	BDE-118	81	79		
PBBz	3	Target	BDE-118	81	79	471.7	
BDE-17	4	Target	BDE-118	81	79	160.8	
BDE-28	5	Target	BDE-118	81	79		
PBEB	6	Target	BDE-118	81	79		
HBBz	7	Target	BDE-118	81	79	471.7	
BDE-49	8	Target	BDE-118	81	79	160.8	
BDE-47	9	Target	BDE-118	81	79	160.8	
BDE-66	10	Target	BDE-118	81	79	160.8	
BDE-77	11	Surrogate	BDE-118	81	79		
BDE-100	12	Target	BDE-118	81	79		
BDE-99	13	Target	BDE-118	81	79		
EH-TBB	14	Target	BDE-118	357	359	79	81
BDE-118	15	Internal		81	79		
BDE-85	16	Target	BDE-118	81	79		
BDE-154	17	Target	BDE-118	81	79		
BDE-153	18	Target	BDE-118	81	79		
BDE-139	19	Target	BDE-118	81	79		
BDE-140	20	Target	BDE-118	81	79		
HBCDD	21	Target	BDE-118	159.8	81	79	
BDE-166	22	Surrogate	BDE-118	81	79		
BDE-183	23	Target	BDE-118	81	79		
BTBPE	24	Target	BDE-181	81	79		
BDE-181	25	Internal		81	79		
BEH-TEBP	26	Target	BDE-181	463.7	461.7	81	79
syn-DP	27	Target	BDE-181	653.8	651.8		
BDE-201	28	Target	BDE-181	408.7	406.7		
anti-DP	29	Target	BDE-181	653.8	651.8		
BDE-197	30	Target	BDE-181	408.7	406.7		
BDE-203	31	Target	BDE-181	81	79		
BDE-208	32	Target	BDE-181	486.8	488.8	81	79
BDE-207	33	Target	BDE-181	486.8	488.8	81	79
BDE-206	34	Target	BDE-181	81	79	486.8	488.8
BDE-209	35	Target	BDE-181	486.8	488.8		
^{13}C-BDE-209	36	Surrogate	BDE-181	494.6	496.6		
DBDPE	37	Target	BDE-181	81	79		

Table S3. Linearity of target analytes and surrogate standards (SS) obtained from the 11-point calibration

FRs	Linearity Range (ng/mL)	R^2
BDE-15	0.0050 - 40	0.9913
BDE-17	0.0050 - 38	0.9958
BDE-28	0.0050 - 40	0.9966
BDE-49	0.010 - 80	0.9975
BDE-47	0.010 - 80	0.9964
BDE-66	0.010 - 80	0.9979
BDE-77 (SS)	0.011 - 86	0.9986
BDE-100	0.010 - 80	0.9967
BDE-99	0.010 - 80	0.9976
BDE-85	0.010 - 80	0.9937
BDE-154	0.021 - 160	0.9934
BDE-153	0.010 - 80	0.9938
BDE-139	0.010 - 80	0.9949
BDE-140	0.010 - 80	0.9961
BDE-166 (SS)	0.012 - 90	0.9945
BDE-183	0.021 - 160	0.9948
BDE-201	0.021 - 160	0.9949
BDE-197	0.021 - 160	0.9951
BDE-203	0.021 - 160	0.9942
BDE-208	0.051 - 400	0.9912
BDE-207	0.051 - 400	0.9915
BDE-206	0.051 - 400	0.9876
BDE-209	0.051 - 400	0.9943
^{13}C-BDE-209	0.0030 - 8.0	0.9977
pTBX	0.0050 - 40	0.9919
PBBz	0.0050 - 40	0.9927
PBEB	0.0050 - 40	0.9944
HBBz	0.0050 - 40	0.9944
EH-TBB	0.010 - 80	0.9300
BTBPE	0.010 - 80	0.9951
BEH-TEBP	0.010 - 80	0.9995
DBDPE	0.10 - 800	0.9971
HBCDD	0.021 - 160	0.9978
syn-DP	0.010 - 80	0.9912
anti-DP	0.010 - 80	0.9899

Table S4. Limit of detection (LOD) for each analyte (calculated from a S/N ratio of 3)

BFRs	LOD (S/N=3)		
	ng/ml	pg/injection	ng/g dw [a]
BDE-15	0.015	0.15	0.03
BDE-17	0.005	0.05	0.01
BDE-28	0.005	0.05	0.01
BDE-49	0.01	0.1	0.02
BDE-47	0.01	0.1	0.02
BDE-66	0.01	0.1	0.02
BDE-100	0.01	0.1	0.02
BDE-99	0.0085	0.085	0.017
BDE-85	0.01	0.1	0.02
BDE-154	0.0078	0.078	0.0156
BDE-153	0.0075	0.075	0.015
BDE-139	0.0091	0.091	0.0182
BDE-140	0.0091	0.091	0.0182
BDE-183	0.013	0.13	0.026
BDE-201	0.0035	0.035	0.007
BDE-197	0.021	0.21	0.042
BDE-203	0.021	0.21	0.042
BDE-208	0.051	0.51	0.102
BDE-207	0.051	0.51	0.102
BDE-206	0.051	0.51	0.102
BDE-209	0.01	0.1	0.02
pTBX	0.0025	0.025	0.005
PBBz	0.0025	0.025	0.005
PBEB	0.005	0.05	0.01
HBBz	0.005	0.05	0.01
EH-TBB	0.015	0.15	0.03
BTBPE	0.01	0.1	0.02
BEH-TEBP	0.005	0.05	0.01
DBDPE	0.14	1.4	0.28
HBCDD	0.04	0.4	0.08
syn-DP	0.0025	0.025	0.005
anti-DP	0.003	0.03	0.006

[a] 0.5 g sample (dw) was assumed to calculate the method LODs.

Table S5. Recoveries of surrogate (internal) standards in blanks, matrix spikes, and food samples

Surrogate standards	Blanks (n=9)	Matrix spikes (n=9)	Samples (n=42)
BDE-77	84%-107%	77%-96%	65%-113%
BDE-166	79%-97%	70%-102%	45%-87%
^{13}C-BDE-209	66%-81%	65%-88%	39%-73%

Table S6. Recoveries of target analytes and surrogate (internal) standards in matrix spikes (MS; n=9) [a]

FRs	Theoretical concentration	Recovery (%)		
	ng/mL	Min	Max	Mean±SD
BDE-15	4.0	58	118	73±18
BDE-17	4.0	66	140	85±22
BDE-28	4.0	63	83	73±5.6
BDE-49	4.0	71	90	78±6.2
BDE-47	4.0	72	94	80±7.3
BDE-66	4.0	76	97	87±7.8
BDE-77 (SS)	7.0	77	96	85±5.7
BDE-100	4.0	66	83	76±5.4
BDE-99	4.0	73	89	81±5.2
BDE-85	4.0	73	90	81±5.3
BDE-154	8.0	59	74	68±4.6
BDE-153	8.0	66	78	74±4.0
BDE-166 (SS)	5.0	70	102	87±11
BDE-183	8.0	67	82	76±5.0
BDE-197	8.0	62	79	71±5.4
BDE-207	20	62	82	74±6.4
BDE-206	20	69	87	80±5.7
$^{13}C_{12}$-209 (SS)	4.0	65	88	79±7.7
BDE-209	20	64	84	76±6.6
pTBX	2.5	65	89	74±6.9
PBBz	2.5	68	92	76±7.0
EH-TBB	5.0	70	133	92±17
BEH-TEBP	5.0	72	94	84±7.4
HBCDD	10	82	126	100±14
syn-DP	5.0	75	98	87±7.4
anti-DP	5.0	76	98	88±7.3

[a] Matrix spikes were analyzed here as part of the QA/QC procedure because standard reference materials for BFR analyses in food items are currently not available for purchase.

Table S7. HFR concentrations in US food items (pg/g ww)

HFRs	Detection frequency	Max	Min	Average
BDE-15	83%	620	n.d.	98
BDE-17	71%	500	n.d.	39
BDE-28	79%	130	n.d.	20
BDE-49	52%	40	n.d.	8.8
BDE-47	33%	58	n.d.	6.4
BDE-66	12%	13	n.d.	0.94
BDE-100	76%	26	n.d.	6.7
BDE-99	45%	80	n.d.	7.6
BDE-85	21%	10	n.d.	1.3
BDE-154	88%	51	n.d.	13
BDE-153	60%	20	n.d.	4.2
BDE-139	33%	40	n.d.	6.2
BDE-140	48%	83	n.d.	11
BDE-183	36%	15	n.d.	2.0
BDE-201	10%	7.7	n.d.	0.51
BDE-197	10%	21	n.d.	1.1
BDE-203	21%	8.7	n.d.	0.46
BDE-208	0%	0	n.d.	n.d.
BDE-207	10%	54	n.d.	4.2
BDE-206	10%	34	n.d.	1.8
BDE-209	60%	200	n.d.	16
**Σ_{21}PBDEs**		**1,100**	**49**	**250**
pTBX	40%	83	n.d.	6.1
PBBz	79%	25	n.d.	3.5
PBEB	19%	110	n.d.	3.7
HBBz	21%	30	n.d.	2.2
EH-TBB	36%	57	n.d.	6.9
BTBPE	14%	12	n.d.	1.1
BEH-TEBP	33%	72	n.d.	4.2
DBDPE	17%	200	n.d.	18
**Σ_8NBFRs**		**270**	**0.10**	**46**
**HBCDD**	**17%**	**260**	**n.d.**	**17**
syn-DP	38%	12	n.d.	0.84
anti-DP	33%	23	n.d.	1.8
**Σ_2DPs**		**28**	**n.d.**	**2.7**
**Σ_{32}HFRs**		**1,400**	**54**	**310**

Table S8. Concentrations of PBDEs (pg/g ww in parentheses) in food items from different countries (ng/g lw)

Country	Sampling time	Sample type	BDE-209	ΣPBDEs	References
UK	2020-2021	meat	0.49 (24) [a]	1.3 (66) [a]	3
		fish	3.5 (32) [a]	7.7 (210) [a]	
		eggs	0.36 (23) [a]	4.5 (310) [a]	
		cheese	0.15 (42) [a]	1.2 (310) [a]	
UK	2015	meat	0.28-0.63 (21-32) [a]	2.4-21 (520-1000) [a]	4
		fish	0.34-1.7 (32-150) [a]	14-40 (400-2800) [a]	
		eggs	0.53 (56) [a]	2.4 (250) [a]	
		cheese	0.21 (37) [a]	5.3 (940) [a]	
UK	2013	meat		(40-590 [c])	5
		fish		(170-8900 [c])	
		egg		(60-450 [c])	
		dairy products		(20-260 [c])	
Latvia	2016-2019	meat	(12 [a] / 9.6 [b])	(34 [a] / 22 [b])	6
		fish	(23 [a] / 7.5 [b])	(620 [a] / 390 [b])	
		eggs	(34 [a] / 24 [b])	(55 [a] / 36 [b])	
		cheese	(2.9 [a] / 2.9 [b])	(21 [a] / 8.5 [b])	
China	2013	meat	20 (3300) [a] / 13 (440) [b]	21 (3400) [a] / 14 (460) [b]	7
		fish/seafood	13 (1300) [a] / 5.5 (210) [b]	14 (1400) [a] / 6.3 (270) [b]	
		eggs	13 (1600) [a] / 11 (1600) [b]	13 (1700) [a] / 12 (1800) [b]	
		dairy products	5.5 (90) [a] / 3.0 (58) [b]	5.9 (96) [a] / 3.2 (66) [b]	
China [d]	2013	eggs		620-46000 [c]	8
Netherlands	2009-2014	meat		0.060-0.58 [a] / 0.040-0.34 [b]	9
		eggs		0.22 [a] / 0.14 [b]	
		fish		(2.5-1300 [a])	
France	2014-2016	meat	(11 [b])	(170 [a])	10
		fish	(5.7 [b])	(540 [a])	
		egg	(22 [b])	(120 [a])	
France	2007-2009	meat	(52 [a])	(540 [a])	11
		fish	(43 [a])	(79 [a])	
		eggs	(49 [a])	(68 [a])	
		cheese	(40 [a])	(65 [a])	
Belgium	2015-2016	meat		(690 [a])	12
		fish		(370 [a])	
		eggs		(210 [a])	
		dairy products		(600 [a])	
Belgium	2006-2007	egg	2.0-7.1 [a]	3.5-7.8 [a]	13
Ireland	2015	fish	(<2.0-60 [c])	(20-1400 [c])	14
		eggs	(<190-800 [c])	(30-960 [c])	
Spain		fish/shellfish	(<20 [c])	(400-1300 [c])	15
Tanzania	2012	eggs		1.1-350 [c]	16
Japan	2011	fish		(2.0-880 [c])	17
US	2001-2002	fish		(56-3300 [a])	18
US	2009	meat		(14-210 [c])	19
		fish		(25-1500 [c])	

		eggs		(89 [c])	
		dairy products		(11-6200 [c])	
US	2013	formula [e]	(42 [a] / 26 [b])	(130 [a] / 42 [b])	[20]
		cereal [e]	(47 [a] / 14 [b])	(38 [a] / 13 [b])	
		puree [e]	(42 [a] / 41 [b])	(190 [a] / 100 [b])	

[a] Mean concentrations

[b] Median concentrations

[c] Ranges

[d] E-waste recycling sites

[e] Baby food

Table S9. Concentrations of NBFRs (pg/g ww in parentheses) in food items from different countries (ng/g lw)

Country	Sampling time	Sample type	pTBX	PBBz	PBT	PBEB	HBBz	EH-TBB	BTBPE	BEH-TEBP	DBDPE	References
UK	2020-2021	meat		0.026 (1.2) [a]	<LOD [c]	<LOD [c]	<LOD [c]	0.63 (43) [a]	4.3 (190) [a]	8.5 (510) [a]	0.38 (18) [a]	[3]
		fish		<LOD [c]	<LOD [c]	<LOD [c]	<LOD [c]	3.3 (17) [a]	22 (200) [a]	20 (390) [a]	1.2 (45) [a]	
		eggs		<LOD [c]	<LOD [c]	<LOD [c]	<LOD [c]	<LOD [c]	36 (2,300) [a]	2.0 (130) [a]	<LOD [a]	
		cheese		<LOD [c]	<LOD [c]	<LOD [c]	<LOD [c]	<LOD [c]	0.39 (97) [a]	0.52 (140) [a]	<LOD [a]	
UK	2015	meat		<LOD [c]	<LOD [c]	<LOD [c]	<LOD [c]	0.19-1.4 [a]	<0.040-2.4 [a]	0.20-0.57 [a]	<1.1-4.6 [a]	[4]
		fish		<LOD [c]	<LOD [c]	<LOD [c]	<LOD [c]	0.22-0.43 [a]	<0.040-0.78 [a]	<0.10-1.1 [a]	<0.63-21 [a]	
		eggs		<LOD [c]	<LOD [c]	<LOD [c]	<LOD [c]	0.10 [a]	0.18 [a]	1.8 [a]	<1.2 [a]	
		cheese		<LOD [c]	<LOD [c]	<LOD [c]	<LOD [c]	0.11 [a]	0.20 [a]	0.22 [a]	<0.74 [a]	
Latvia	2016-2019	meat				(0.34-5.7 [c])	(1.4-9.8 [c])					[6]
		fish				(<0.96-3.1 [c])	(2.8-18 [c])					
		eggs				(<0.43-1.8 [c])	(0.93-5.7 [c])					
		cheese				(<0.30-2.9 [c])	(1.9-12 [c])					
France	2014-2016	meat		(<LOD-18 [c])	(<LOD-7.0 [c])	(<LOD-0.071 [c])	(<LOD-150 [c])	(<LOD-12 [c])	(<LOD-15 [c])			[10]
		fish		(<LOD-6.4 [c])	(<LOD-20 [c])	(<LOD-0.17 [c])	(<LOD-38 [c])	(<LOD-10 [c])	(<LOD-10 [c])			
		egg		(<LOD-4.0 [c])	(<LOD-12 [c])	(<LOD-0.13 [c])	(<LOD-16 [c])	(<LOD-1.3 [c])	(<LOD-12 [c])			
Belgium	2015-2016	meat								(6.0 [a])		[12]
		fish								(<LOD [c])		
		eggs								(<LOD [c])		
		dairy products								(<LOD [c])		
Spain		fish/shellfish				(<20 [c])	(<20-200 [c])				(<10 [c])	[15]
China [d]	2010	eggs						(<LOD-100 [c])		(<LOD-1800 [c])		[21]
China	2010	eggs						(<LOD [c])		(<LOD-540 [c])		[21]
China [d]	2013	eggs					2.0-6.8 [c]		0.40-78 [c]		4.5-190 [c]	[8]
China	2011	meat			0.026-0.16 [c]		<LOD-0.16 [c]		<LOD-0.067 [c]		<LOD-23 [c]	[22]
		fish			0.030-0.37 [c]		<LOD-2.2 [c]		<LOD-1.9 [c]		<LOD-70 [c]	
		eggs			0.0080-0.10 [c]		<LOD-0.076 [c]		<LOD-4.1 [c]		<LOD-20 [c]	
China [d]	2011	fish				<0.17 [a]	6.5 [a]	62 [a]	6.8 [a]	15 [a]	<0.45 [a]	[23]
		meat				<0.17-0.87 [a]	0.41-0.56 [a]	24-38 [a]	1.5-5.4 [a]	7.2-12 [a]	<0.45 [a]	

		eggs				<0.17 [a]	<0.15 [a]	3.1-5.4 [a]	<0.35-3.8 [a]	0.88-1.6 [a]	<0.45 [a]	
China	2011	fish				<0.17 [a]	<0.15 [a]	4.0 [a]	2.1 [a]	1.9 [a]	<0.45 [a]	23
		meat				<0.17-1.2 [a]	<0.15 [a]	2.1-2.7 [a]	<0.35-2.7 [a]	<0.25-1.8 [a]	<0.45 [a]	
		eggs				<0.17 [a]	<0.15 [a]	1.2-1.7 [a]	<0.35 [a]	<0.25 [a]	<0.45 [a]	
China	2018	meat	0.038 [a]	<LOD [a]	0.010 [a]		0.13 [a]		<LOD [a]			24
		poultry	0.032 [a]	<LOD [a]	<LOD [a]		0.017 [a]		<LOD [a]			
		aquatic food	<LOD [a]	0.091 [a]	0.021 [a]		0.049 [a]		<LOD [a]			
		eggs	<LOD [a]	<LOD [a]	<LOD [a]		0.016 [a]		<LOD [a]			
		dairy products	0.022 [a]	<LOD [a]	<LOD [a]		0.0070 [a]		<LOD [a]			
Tanzania	2012	eggs							<LOD-9.8 [c]			16
US	2013	formula [e]		(1.3 [a])			(6.2 [a] / 6.2 [b])				(19 [a] / 21 [b])	20
		cereal [e]		<LOD			(2.4 [a] / 1.4 [b])				(18 [a] / 11 [b])	
		puree [e]		<LOD			(5.6 [a] / 1.0 [b])				<LOD	

[a] Mean concentrations

[b] Median concentrations

[c] Ranges

[d] E-waste recycling sites

[e] baby food

Table S10. Concentrations of HBCDDs (pg/g ww in parentheses) in food items from different countries (ng/g lw)

Country	Sampling time	Sample type	ΣHBCDDs	References
UK	2020-2021	meat	0.16 (8.9) [a]	3
		fish	0.83 (67) [a]	
		eggs	0.19 (16) [a]	
		cheese	0.050 (12) [a]	
UK	2015	meat	0.32-4.6 (26-200) [a]	4
		fish	3.6-16 (180-830) [a]	
		eggs	1.3 (300) [a]	
		cheese	<0.24 (<43) [a]	
UK	2013	meat	(40 [a])	5
		fish	(1400 [a])	
		egg	(<20 [a])	
		dairy products	(40 [a])	
Latvia	2016-2019	meat	(270 [a] / 160 [b])	6
		fish	(730 [a] / 480 [b])	
		eggs	(24 [a] / 16 [b])	
		cheese	(330 [a] / 160 [b])	
China	2013	meat	2.8 (570) [a] / 2.4 (180) [b]	7
		fish/seafood	7.0 (410) [a] / 5.6 (290) [b]	
		eggs	4.5 (600) [a] / 4.0 (500) [b]	
		dairy products	1.4 (140) [a] / 1.1 (44) [b]	
China [d]	2013	eggs	56-7600 [c]	8
China [d]	2011	fish	310 [a]	23
		meat	<0.060-79 [a]	
		eggs	2.2-47 [a]	
China	2011	fish	5.9 [a]	23
		meat	0.090-3.1 [a]	
		eggs	0.50-1.70 [a]	
France	2014-2016	meat	(130 [a])	10
		fish	(140 [a])	
France	2007-2009	meat	(130 [a])	11
		fish	(140 [a])	
		eggs	(26 [a])	
		cheese	(20 [a])	
Belgium	2015-2016	meat	(43 [a])	12
		fish	(130 [a])	
		eggs	(970 [a])	
		dairy products	(0.10 [a])	
Belgium	2006-2007	egg	6.5-8.5 [a]	13
Ireland	2015	fish	(70-410 [c])	14
		eggs	(10-20 [c])	
Tanzania	2012	eggs	<LOD-63 [c]	16

Japan	2011	fish	(<LOD-22000 [c])	[17]
Sweden	1999	meat	<1.0-6.5 [a]	[25]
		fish	48 [a]	
		egg	9.4 [a]	
US	2009	meat	(23-190 [c])	[19]
		fish	(<29-590 [c])	
		eggs	(<11 [c])	
		dairy products	(<64 [c])	

[a] Mean concentrations

[b] Median concentrations

[c] Ranges

[d] E-waste recycling sites

Table S11. Concentrations of DPs (pg/g ww in parentheses) in food items from different countries (ng/g lw)

Country	Sampling time	Sample type	*syn*-DP	*anti*-DP	ΣDPs	f_{anti}	References
Lebanon	2017	meat	(10-12 [a])	(7.4-8.1 [a])	(17-20 [a])	0.26-0.76	[26]
		fish	(2.0-4.2 [a])	(3.0-3.9 [a])	(5.0-8.0 [a])	0.26-0.76	
		egg	(1.7-3.1 [a])	(5.2-5.8 [a])	(6.9-8.9 [a])	0.26-0.76	
		dairy products	(1.0-2.0 [a])	(0.70-1.1 [a])	(1.7-3.1 [a])	0.26-0.76	
Latvia	2016-2019	meat	(0.77-4.7 [c])	(1.5-11 [c])	(2.3-16 [c])	0.63-0.81	[6]
		fish	(2.7-7.7 [c])	(4.8-14 [c])	(7.5-21 [c])	0.56-0.77	
		eggs	(2.3-20 [c])	(8.1-54 [c])	(10-75 [c])	0.66-0.78	
		cheese	(0.50-3.9 [c])	(2.1-14 [c])	(2.6-18 [c])	0.67-0.80	
China [d]	2013	eggs			30-2200 [c]	0.74-0.76	[8]
Belgium	2015-2016	meat			(10 [a])	0.80	[12]
		fish			(<LOD [a])		
		eggs			(160 [a])	0.80	
		dairy products			(9.0 [a])	0.80	
Japan	2011	fish	(<LOD-6.1 [c])	(<LOD-8.1 [c])	(<LOD-14 [c])	0.56-0.72	[17]
Japan	2012	meat & eggs	(1.0)	(0.90)	(1.9)	0.47	[27]
		fish	(0.60)	(0.90)	(1.5)	0.60	
		dairy products	<LOD	<LOD	<LOD		

[a] Mean concentrations

[b] Median concentrations

[c] Ranges

[d] E-waste recycling sites

Table S12. Median concentrations (pg/g ww) of PBDEs and NBFRs in food items by price range and ANOVA test results

Price	Number of individual samples included	Number of composite samples analyzed	Σ_{13}PBDEs	Σ_4NBFRs
low	18	11	170	26
medium	27	17	180	4.5
high	27	14	170	9.8
F value (ANOVA)	-	-	0.187	3.202
Sig. (ANOVA)	-	-	0.830	0.052

Table S13. Median concentrations (pg/g ww) of PBDEs and NBFRs in organic and non-organic US food items and Paired-Samples t test results

Food category	Number of individual samples included	Number of composite samples analyzed	Σ_{13}PBDEs	Σ_4NBFRs
organic				
beef	1	1	170	1.5
pork	5	2	190	1.2
chicken	8	4	120	21
turkey	3	2	280	17
cheese	3	3	450	26
egg	6	2	130	18
sum	26	14	190	18
non-organic				
beef	8	3	94	4.4
pork	4	2	170	40
chicken	3	2	190	17
turkey	2	2	190	38
cheese	3	3	530	3.3
egg	9	3	180	4.5
sum	29	15	180	5.9
p value	-	-	0.941	0.735

Table S14. Average daily consumption of food items (g/day) [a] for US children (< 20 years old) and adults (≥ 20 years old)[1]

Age	Gender	Beef	Pork	Chicken	Turkey	Salmon [b]	Cod [b]	Catfish [b]	Tilapia [b]	Tuna [b]	Cheese	Egg
< 20 years	Male	54	32	55	8.0	0.96	0.96	0.96	0.96	0.96	26	19
	Female	35	21	49	7.1	0.81	0.81	0.81	0.81	0.81	23	15
	All	45	27	52	7.6	0.89	0.89	0.89	0.89	0.89	25	17
≥ 20 years	Male	82	58	72	13	3.7	3.7	3.7	3.7	3.7	32	32
	Female	44	31	54	11	2.3	2.3	2.3	2.3	2.3	24	21
	All	63	44	63	12	3.0	3.0	3.0	3.0	3.0	28	26

[a] US per capita loss-adjusted food availability; unit is converted from lb/year to g/day.

[b] Consumption of salmon (cod/catfish/tilapia/tuna) is assumed to be total consumption of fish divided by 5.

Table S15. Average daily consumption of food items (g/day) [a] for US adults (≥ 20 years old) of different races[1]

Race	Beef	Pork	Chicken	Turkey	Salmon [b]	Cod [b]	Catfish [b]	Tilapia [b]	Tuna [b]	Cheese	Egg
Hispanic [c]	60	34	62	6.5	2.3	2.3	2.3	2.3	2.3	22	26
Non-Hispanic white	60	40	57	11	2.2	2.2	2.2	2.2	2.2	30	23
Non-Hispanic black	54	39	78	15	2.7	2.7	2.7	2.7	2.7	22	24
Non-Hispanic Asian	39	43	57	7.6	4.3	4.3	4.3	4.3	4.3	17	23

[a] US per capita loss-adjusted food availability; unit is converted from lb/year to g/day.

[b] Consumption of salmon (cod/catfish/tilapia/tuna) is assumed to be total consumption of fish divided by 5.

[c] Includes Mexican-American persons.

Table S16. Average body weight (kg) of US children (< 20 years old) and adults (≥ 20 years old)[2]

Age	Male	Female	All
< 20 years	37.8	35.9	36.9
≥ 20 years	90.6	77.5	83.9

Table S17. Average body weight (kg) of US adults (≥ 20 years old) by race[2]

Race	Male	Female	All
Hispanic [a]	87.9	76.2	81.6
Non-Hispanic white	92.2	77.5	84.9
Non-Hispanic black	90.8	85.5	88.0
Non-Hispanic Asian	76.2	61.2	68.5

[a] Includes Mexican-American persons.

Table S18. Estimated median dietary intake of HFRs (ng/kg bw/day) [a] for US children and adults

HFRs	Children (< 20 years old)			Adults (20 + years old)		
	**Boys**	**Girls**	**All**	**Men**	**Women**	**All**
BDE-15	0.37	0.30	0.34	0.23	0.17	0.21
BDE-17	0.12	0.10	0.11	0.068	0.055	0.062
BDE-28	0.042	0.036	0.039	0.030	0.023	0.027
BDE-49	0.031	0.026	0.029	0.019	0.015	0.017
BDE-47	0.0036	0.0033	0.0035	0.0038	0.0030	0.0035
BDE-100	0.030	0.025	0.028	0.019	0.014	0.017
BDE-99	0.016	0.013	0.015	0.011	0.0086	0.0097
BDE-154	0.064	0.054	0.060	0.039	0.030	0.035
BDE-153	0.021	0.018	0.020	0.013	0.010	0.012
BDE-139	0.041	0.034	0.038	0.024	0.018	0.021
BDE-140	0.021	0.015	0.018	0.015	0.010	0.013
BDE-183	0.0068	0.0055	0.0063	0.0047	0.0034	0.0041
BDE-209	0.036	0.031	0.033	0.023	0.018	0.020
**Σ_{13}PBDEs**	**0.80**	**0.67**	**0.74**	**0.50**	**0.38**	**0.45**
pTBX	0.0082	0.0075	0.0079	0.0063	0.0051	0.0058
PBBZ	0.012	0.0095	0.011	0.0075	0.0056	0.0066
EHTBB	0.0064	0.0044	0.0055	0.0050	0.0032	0.0041
BEHTBP	0.0017	0.0015	0.0016	0.0012	0.0011	0.0012
**Σ_4NBFRs**	**0.028**	**0.023**	**0.026**	**0.020**	**0.015**	**0.018**
syn-DP	0.0012	0.0012	0.0012	0.00088	0.00073	0.00081
anti-DP	0.00034	0.00031	0.00033	0.00046	0.00035	0.00041
**Σ_2DPs**	**0.0016**	**0.0015**	**0.0015**	**0.0013**	**0.0011**	**0.0012**
**Σ_{19}HFRs**	**0.83**	**0.69**	**0.77**	**0.52**	**0.40**	**0.47**

[a] Median concentrations of HFRs in US food items were applied. Only HFRs with a DF exceeding 30% were included.

Table S19. Estimated median dietary intake of HFRs (ng/kg bw/day) [a] for US adults by race

HFRs	Hispanic	Non-Hispanic white	Non-Hispanic black	Non-Hispanic Asian
BDE-15	0.17	0.20	0.18	0.20
BDE-17	0.053	0.062	0.050	0.054
BDE-28	0.023	0.023	0.025	0.032
BDE-49	0.015	0.016	0.016	0.018
BDE-47	0.0026	0.0028	0.0032	0.0049
BDE-100	0.015	0.015	0.016	0.017
BDE-99	0.0090	0.0086	0.0089	0.0099
BDE-154	0.031	0.033	0.031	0.034
BDE-153	0.010	0.011	0.012	0.012
BDE-139	0.021	0.020	0.022	0.021
BDE-140	0.011	0.011	0.011	0.013
BDE-183	0.0035	0.0036	0.0040	0.0048
BDE-209	0.018	0.019	0.020	0.021
**Σ_{13}PBDEs**	**0.39**	**0.42**	**0.39**	**0.44**
pTBX	0.0053	0.0047	0.0060	0.0079
PBBZ	0.0061	0.0061	0.0060	0.0066
EHTBB	0.0033	0.0037	0.0035	0.0052
BEHTBP	0.00087	0.0010	0.0012	0.0012
**Σ_4NBFRs**	**0.016**	**0.015**	**0.017**	**0.021**
syn-DP	0.00076	0.00068	0.00087	0.0011
anti-DP	0.00031	0.00031	0.00038	0.00067
**Σ_2DPs**	**0.0011**	**0.00099**	**0.0012**	**0.0017**
**Σ_{19}HFRs**	**0.40**	**0.44**	**0.41**	**0.46**

[a] Median concentrations of HFRs in US food items were applied. Only HFRs with a DF exceeding 30% were included.

Table S20. Reference dose (RfD) values (ng/kg bw/day) for HFRs

BFRs	RfD	References
BTBPE	243,000	[28]
EH-TBB	20,000	[28]
BEH-TEBP	20,000	[28]
DBDPE	333,333	[28]
BDE-209	7,000 [a]	[29]
Octa-BDE	3,000	[29]
Penta-BDE	2,000	[29]
BDE-47	100	[29]
BDE-99	100	[29]
BDE-153	200	[29]
∑HBCDDs	200,000	[30]
∑DPs	5,000,000	[31]

[a] For decaBDE-209, EPA has assigned an oral slope factor for carcinogenic risk of 700 $(ng/kg\ bw/day)^{-1}$ (EPA IRIS 2008).

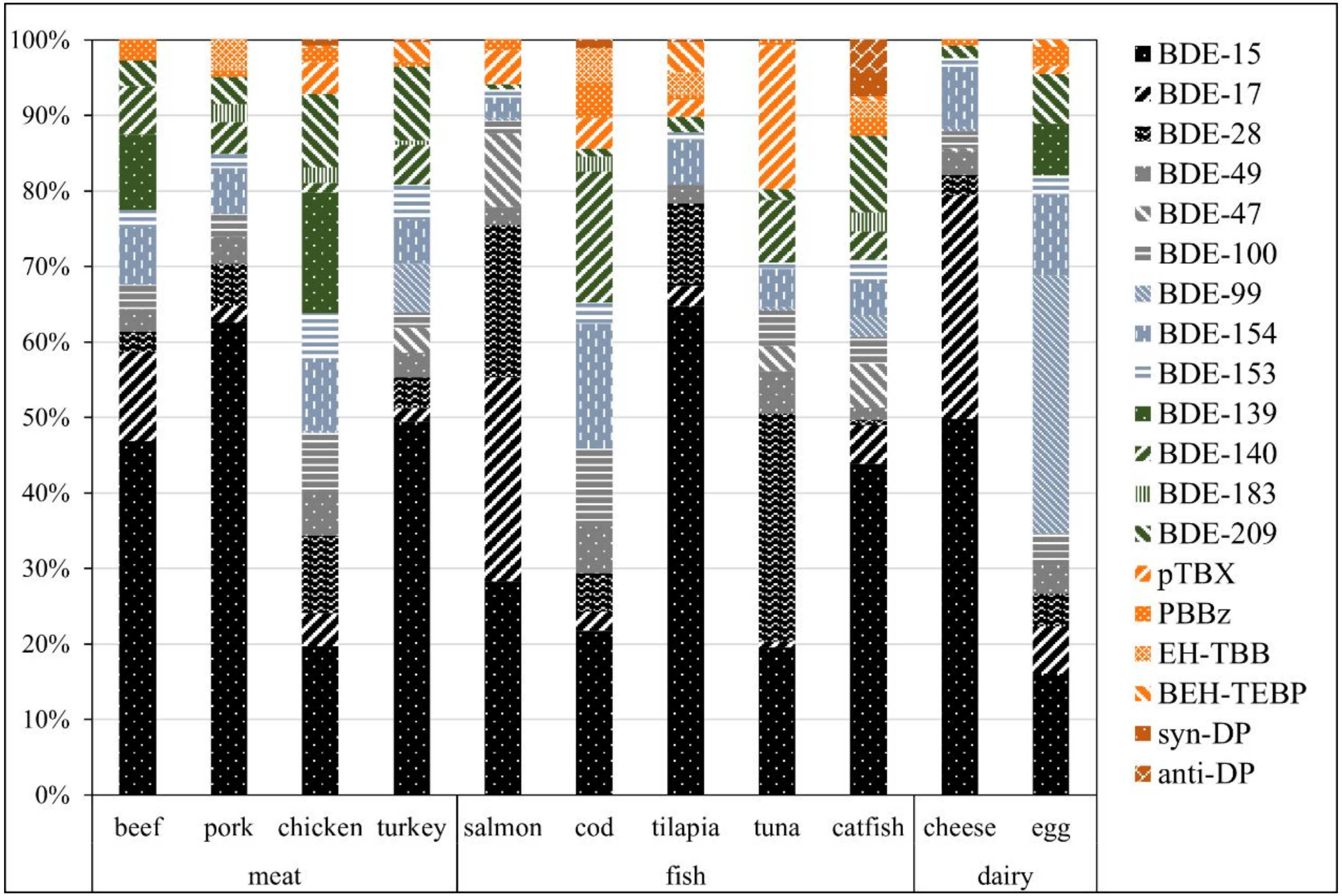

Fig. S1 Relative contribution of HFRs in different US food items. Median concentrations were used. Only HFRs with a DF exceeding 30% were included.

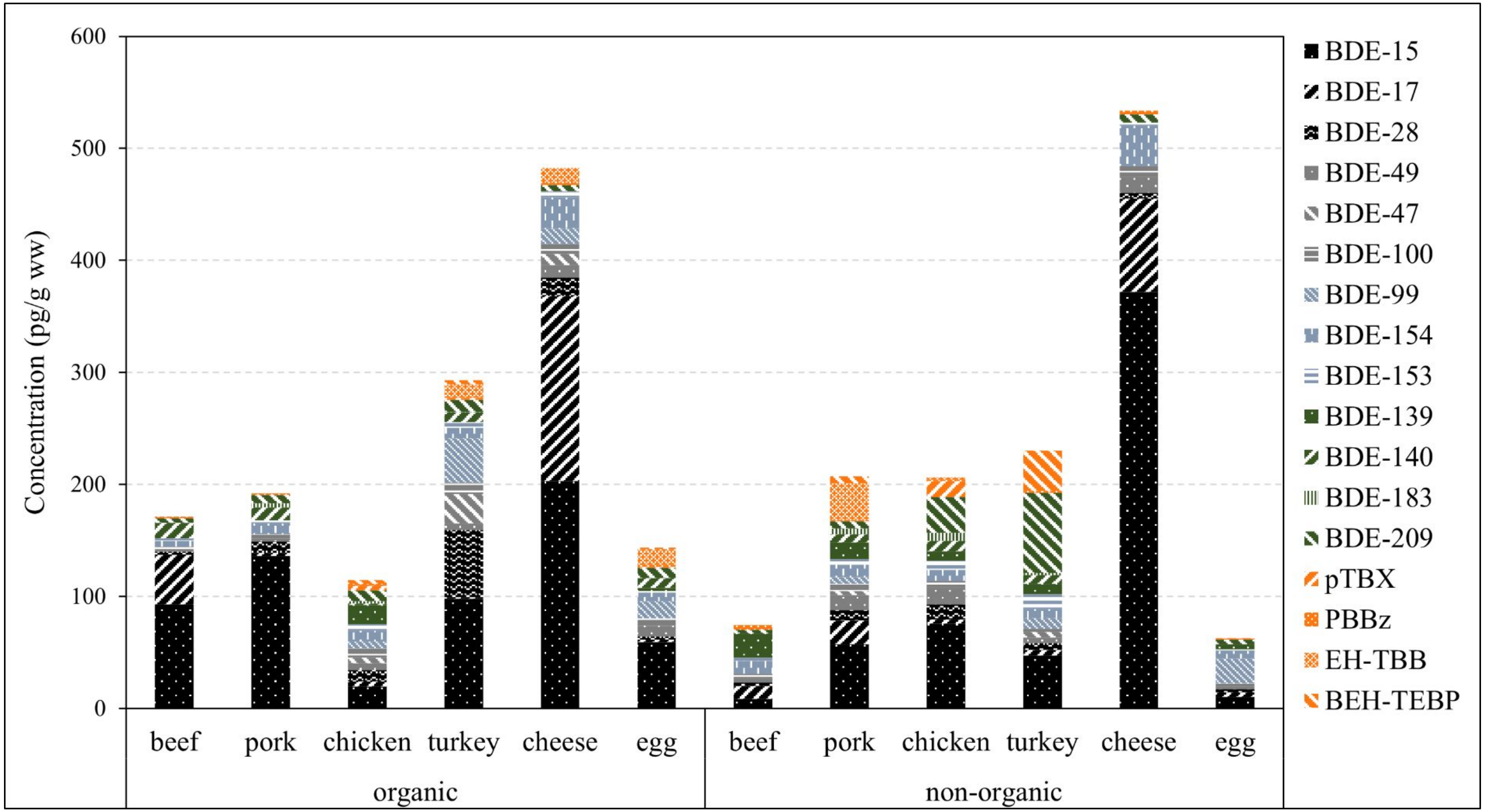

Fig. S2 Median concentrations of PBDEs and NBFRs in organic and non-organic US food items. Only PBDEs and NBFRs with a DF exceeding 30% are included.

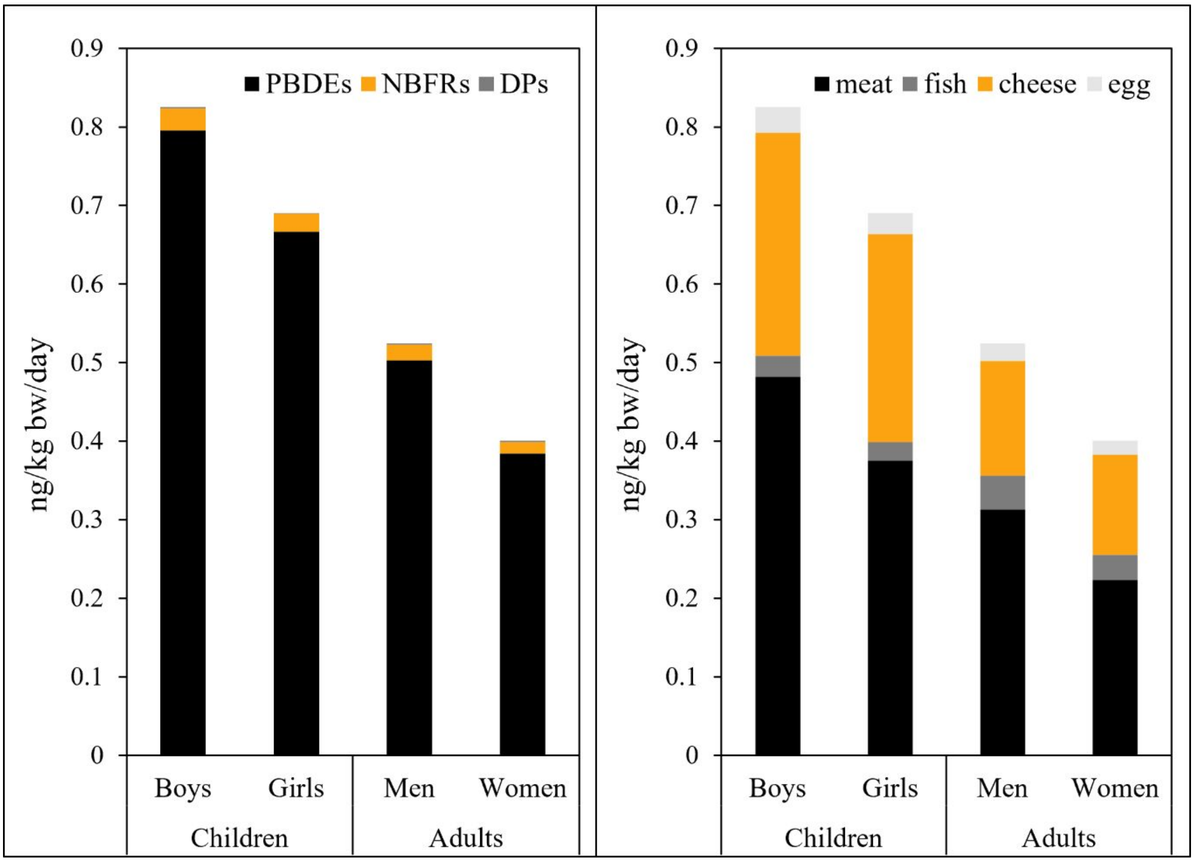

Fig. S3 Estimated median dietary intake of HFRs for US children (< 20 years old) and adults (≥ 20 years old): contribution of different FR classes (left) and food categories (right). See Table S18 for detailed data.

References

(1) US Department of Agriculture Economic Research Service (USDA ERS). Commodity Consumption by Population Characteristics. **2020.** Available at: https://www.ers.usda.gov/data-products/commodity-consumption-by-population-characteristics/ (accessed 2022-07).

(2) Fryar, C.; Carroll, M.; Gu, Q.; Afful, J.; Ogden, C. Anthropometric reference data for children and adults: United States, 2015–2018. National Center for Health Statistics. *Vital Health Stat* **2021,** *3*, 46.

(3) Ma, Y.; Stubbings, W. A.; Abdallah, M. A. E.; Cline-Cole, R.; Harrad, S. Temporal trends in concentrations of brominated flame retardants in UK foodstuffs suggest active impacts of global phase-out of PBDEs and HBCDD. *Sci Total Environ* **2023,** *863*, 160956.

(4) Tao, F.; Abdallah, M. A. E.; Ashworth, D. C.; Douglas, P.; Toledano, M. B.; Harrad, S. Emerging and legacy flame retardants in UK human milk and food suggest slow response to restrictions on use of PBDEs and HBCDD. *Environ Int* **2017,** *105*, 95–104.

(5) Fernandes, A. R.; Mortimer, D.; Rose, M.; Smith, F.; Panton, S.; Garcia-Lopez, M. Bromine content and brominated flame retardants in food and animal feed from the UK. *Chemosphere* **2016,** *150*, 472–478.

(6) Zacs, D.; Perkons, I.; Abdulajeva, E.; Pasecnaja, E.; Bartkiene, E.; Bartkevics, V. Polybrominated diphenyl ethers (PBDEs), hexabromocyclododecanes (HBCDD), dechlorane-related compounds (DRCs), and emerging brominated flame retardants (EBFRs) in foods: The levels, profiles, and dietary intake in Latvia. *Sci Total Environ* **2021,** *752*, 141996.

(7) Wang, J.; Zhao, X.; Wang, Y.; Shi, Z. Tetrabromobisphenol A, hexabromocyclododecane isomers and polybrominated diphenyl ethers in foodstuffs from Beijing, China: Contamination levels, dietary exposure and risk assessment. *Sci Total Environ* **2019,** *666*, 812–820.

(8) Zeng, Y. H.; Luo, X. J.; Tang, B.; Mai, B. X. Habitat- and species-dependent accumulation of organohalogen pollutants in home-produced eggs from an electronic waste recycling site in South China: Levels, profiles, and human dietary exposure. *Environ Pollut* **2016,** *216*, 64–70.

(9) Gebbink, W. A.; van der Lee, M. K.; Peters, R. J. B.; Traag, W. A.; Dam, G. T.; Hoogenboom, R.; van Leeuwen, S. P. J. Brominated flame retardants in animal derived foods in the Netherlands between 2009 and 2014. *Chemosphere* **2019,** *234*, 171–178.

(10) Venisseau, A.; Bichon, E.; Brosseaud, A.; Vaccher, V.; Lesquin, E.; Larvor, F.; Durand, S.;

Dervilly-Pinel, G.; Marchand, P.; Le Bizec, B. Occurence of legacy and novel brominated flame retardants in food and feed in France for the period 2014 to 2016. *Chemosphere* **2018,** *207*, 497–506.

(11) Riviere, G.; Sirot, V.; Tard, A.; Jean, J.; Marchand, P.; Veyrand, B.; Le Bizec, B.; Leblanc, J. C. Food risk assessment for perfluoroalkyl acids and brominated flame retardants in the French population: results from the second French total diet study. *Sci Total Environ* **2014,** *491-492*, 176–183.

(12) Poma, G.; Malysheva, S. V.; Goscinny, S.; Malarvannan, G.; Voorspoels, S.; Covaci, A.; Van Loco, J. Occurrence of selected halogenated flame retardants in Belgian foodstuff. *Chemosphere* **2018,** *194*, 256–265.

(13) Covaci, A.; Roosens, L.; Dirtu, A. C.; Waegeneers, N.; Van Overmeire, I.; Neels, H.; Goeyens, L. Brominated flame retardants in Belgian home-produced eggs: levels and contamination sources. *Sci Total Environ* **2009,** *407* (15), 4387–4396.

(14) Garcia Lopez, M.; Driffield, M.; Fernandes, A. R.; Smith, F.; Tarbin, J.; Lloyd, A. S.; Christy, J.; Holland, M.; Steel, Z.; Tlustos, C. Occurrence of polybrominated diphenylethers, hexabromocyclododecanes, bromophenols and tetrabromobisphenols A and S in Irish foods. *Chemosphere* **2018,** *197*, 709–715.

(15) Trabalon, L.; Vilavert, L.; Domingo, J. L.; Pocurull, E.; Borrull, F.; Nadal, M. Human exposure to brominated flame retardants through the consumption of fish and shellfish in Tarragona County (Catalonia, Spain). *Food Chem Toxicol* **2017,** *104*, 48–56.

(16) Polder, A.; Muller, M. B.; Brynildsrud, O. B.; de Boer, J.; Hamers, T.; Kamstra, J. H.; Lie, E.; Mdegela, R. H.; Moberg, H.; Nonga, H. E.; Sandvik, M.; Skaare, J. U.; Lyche, J. L. Dioxins, PCBs, chlorinated pesticides and brominated flame retardants in free-range chicken eggs from peri-urban areas in Arusha, Tanzania: Levels and implications for human health. *Sci Total Environ* **2016,** *551-552*, 656–67.

(17) Kakimoto, K.; Nagayoshi, H.; Yoshida, J.; Akutsu, K.; Konishi, Y.; Toriba, A.; Hayakawa, K. Detection of Dechlorane Plus and brominated flame retardants in marketed fish in Japan. *Chemosphere* **2012,** *89* (4), 416–419.

(18) Hites, R. A.; Foran, J. A.; Schwager, S. J.; Knuth, B. A.; Hamilton, M. C.; Carpenter, D. O. Global Assessment of Polybrominated Diphenyl Ethers in Farmed and Wild Salmon. *Environ Sci*

Technol **2004,** *38* (19), 4945–4949.

(19) Schecter, A.; Haffner, D.; Colacino, J.; Patel, K.; Papke, O.; Opel, M.; Birnbaum, L. Polybrominated diphenyl ethers (PBDEs) and hexabromocyclodecane (HBCD) in composite U.S. food samples. *Environ Health Perspect* **2010,** *118* (3), 357–362.

(20) Liu, L. Y.; Salamova, A.; Hites, R. A. Halogenated flame retardants in baby food from the United States and from China and the estimated dietary intakes by infants. *Environ Sci Technol* **2014,** *48* (16), 9812–9818.

(21) Zheng, X.; Xu, F.; Luo, X.; Mai, B.; Covaci, A. Phosphate flame retardants and novel brominated flame retardants in home-produced eggs from an e-waste recycling region in China. *Chemosphere* **2016,** *150*, 545–550.

(22) Shi, Z.; Zhang, L.; Li, J.; Zhao, Y.; Sun, Z.; Zhou, X.; Wu, Y. Novel brominated flame retardants in food composites and human milk from the Chinese Total Diet Study in 2011: Concentrations and a dietary exposure assessment. *Environ Int* **2016,** *96*, 82–90.

(23) Labunska, I.; Abdallah, M. A.-E.; Eulaers, I.; Covaci, A.; Tao, F.; Wang, M.; Santillo, D.; Johnston, P.; Harrad, S. Human dietary intake of organohalogen contaminants at e-waste recycling sites in Eastern China. *Environ Int* **2015,** *74*, 209–220.

(24) Jian, K.; Zhao, L.; Ya, M.; Zhang, Y.; Su, H.; Meng, W.; Li, J.; Su, G. Dietary intake of legacy and emerging halogenated flame retardants using food market basket estimations in Nanjing, eastern China. *Environ Pollut* **2020,** *258*, 113737.

(25) Remberger, M.; Sternbeck, J.; Palm, A.; Kaj, L.; Strömberg, K.; Brorström-Lundén, E. The environmental occurrence of hexabromocyclododecane in Sweden. *Chemosphere* **2004,** *54* (1), 9–21.

(26) Abdel Malak, I.; Cariou, R.; Guiffard, I.; Venisseau, A.; Dervilly-Pinel, G.; Jaber, F.; Le Bizec, B. Assessment of Dechlorane Plus and related compounds in foodstuffs and estimates of daily intake from Lebanese population. *Chemosphere* **2019,** *235*, 492–497.

(27) Kakimoto, K.; Nagayoshi, H.; Takagi, S.; Akutsu, K.; Konishi, Y.; Kajimura, K.; Hayakawa, K.; Toriba, A. Inhalation and dietary exposure to Dechlorane Plus and polybrominated diphenyl ethers in Osaka, Japan. *Ecotoxicol Environ Saf* **2014,** *99*, 69–73.

(28) Ali, N.; Dirtu, A. C.; Van den Eede, N.; Goosey, E.; Harrad, S.; Neels, H.; t Mannetje, A.; Coakley, J.; Douwes, J.; Covaci, A. Occurrence of alternative flame retardants in indoor dust from

New Zealand: indoor sources and human exposure assessment. *Chemosphere* **2012,** *88* (11), 1276–1282.

(29) US Environmental Protection Agency (US EPA). Technical Fact Sheet - Polybrominated Diphenyl Ethers (PBDEs) (EPA 505-F-17-015). **2017**. Available at: https://www.epa.gov/sites/default/files/2014-03/documents/ffrrofactsheet_contaminant_perchlorate_january2014_final_0.pdf (accessed 2022-09)

(30) Pakalin, S.; Cole, T.; Steinkellner, J.; Nicolas, R.; Tissier, C.; Munn, S.; Eisenreich, S. J. E. R. E. Review on production processes of decabromodiphenyl ether (decaBDE) used in polymeric applications in electrical and electronic equipment, and assessment of the availability of potential alternatives to decaBDE. **2007,** *22693*, 47.

(31) Wang, D. G.; Alaee, M.; Byer, J. D.; Brimble, S.; Pacepavicius, G. Human health risk assessment of occupational and residential exposures to dechlorane plus in the manufacturing facility area in China and comparison with e-waste recycling site. *Sci Total Environ* **2013,** *445–446*, 329–336.
